# Genomic analyses of *Neisseria gonorrhoeae* reveal an association of the gonococcal genetic island with antimicrobial resistance

**DOI:** 10.1016/j.jinf.2016.08.010

**Published:** 2016-12

**Authors:** Odile B. Harrison, Marianne Clemence, Joseph P. Dillard, Christoph M. Tang, David Trees, Yonatan H. Grad, Martin C.J. Maiden

**Affiliations:** a Department of Zoology, University of Oxford, Oxford, UK; b Department of Medical Microbiology, University of Wisconsin-Madison School of Medicine and Public Health, Madison, WI, USA; c Sir William Dunn School of Pathology, University of Oxford, Oxford, UK; d Centers for Disease Control and Prevention, Atlanta, GA, USA; e Harvard TH Chan School of Public Health, Boston, MA, USA; f Division of Infectious Diseases, Brigham and Women's Hospital, Harvard Medical School, Boston, MA, USA

**Keywords:** Whole-genome sequencing, Antimicrobial resistance, Type IV secretion system, Gene-by-gene annotation

## Abstract

**Objectives:**

Antimicrobial resistance (AMR) threatens our ability to treat the sexually transmitted bacterial infection gonorrhoea. The increasing availability of whole genome sequence (WGS) data from *Neisseria gonorrhoeae* isolates, however, provides us with an opportunity in which WGS can be mined for AMR determinants.

**Methods:**

Chromosomal and plasmid genes implicated in AMR were catalogued on the PubMLST *Neisseria* database (http://pubmlst.org/neisseria). AMR genotypes were identified in WGS from 289 gonococci for which MICs against several antimicrobial compounds had been determined. Whole genome comparisons were undertaken using whole genome MLST (wgMLST).

**Results:**

Clusters of isolates with distinct AMR genotypes were apparent following wgMLST analysis consistent with the occurrence of genome wide genetic variation. This included the presence of the gonococcal genetic island (GGI), a type 4 secretion system shown to increase recombination and for which possession was significantly associated with AMR to multiple antimicrobials.

**Conclusions:**

Evolution of the gonococcal genome occurs in response to antimicrobial selective pressure resulting in the formation of distinct *N. gonorrhoeae* populations evidenced by the wgMLST clusters seen here. Genomic islands offer selective advantages to host bacteria and possession of the GGI may, not only facilitate the spread of AMR in gonococcal populations, but may also confer fitness advantages.

## Highlights

• Gene-by-gene annotation of WGS combined with whole genome MLST (wgMLST) analyses.
• Provision of an online catalogue of gonococcal antimicrobial resistance (AMR) genes.
• Gene-by-gene analysis found an association of AMR with a T4SS known to enhance HGT.
• The T4SS may facilitate the spread of AMR but may also confer fitness advantages.

## Introduction

*Neisseria gonorrhoeae*, the aetiological agent of the sexually transmitted disease gonorrhoea, annually causes an estimated 108 million cases globally.^1^ Untreated gonorrhoea can result in severe sequelae including pelvic inflammatory disease, infertility, neonatal conjunctivitis as well as disseminated gonococcal infections. Gonorrhoea may also lead to increased HIV transmission.^2^ While effective treatment of gonorrhoea is a priority for public health globally, treatment options have diminished as *N. gonorrhoeae* strains have developed resistance to multiple classes of antibiotics.^3^

Gonococci become resistant to antibiotics through spontaneous mutation and/or horizontal genetic transfer (HGT) with resistance conferred through all known mechanisms including antimicrobial inactivation, antimicrobial target alteration as well as increased export and decreased uptake of antimicrobial compounds.^2^ For example, resistance to fluoroquinolones, which inhibit the action of topoisomerase enzymes involved in DNA replication, occurs through amino acid alterations in the chromosomal DNA gyrase gene, *gyrA* and/or the DNA topoisomerase gene, *parC*.^4^ The penicillin binding proteins 1 and 2 (PBP1 and PBP2) encoded by *ponA* and *penA* respectively are essential in the final stages of peptidoglycan synthesis involved in cell wall assembly. Beta-lactams such as penicillin and cephalosporin target PBP1 and PBP2 inhibiting cell wall synthesis; however, non-synonymous mutations combined with recombination alter the antibiotic target thereby limiting beta-lactam activity.^5^ Resistance to spectinomycin and azithromycin, which both interfere with protein synthesis, occurs through point mutations in the nucleotide sequences encoding either 16S rRNA or 23S rRNA respectively.^6^, ^7^ Increased export of antimicrobial compounds may occur through alterations of the *mtrR* efflux pump repressor gene and/or its associated promoter resulting in over-expression of the MtrCDE efflux pump,^8^, ^9^ while decreased antimicrobial uptake occurs through alteration of the major outer membrane protein PorB encoded by *porB1b* (also known as *penB*).^10^ Finally, antimicrobial inactivation may result from plasmid-mediated beta-lactamases and/or *tetM* genes which facilitate penicillin and/or tetracycline resistance.^11^, ^12^

Advances in sequencing and bioinformatics technology provide rapid and automated analysis of whole genome sequence data (WGS) and understanding antimicrobial resistance (AMR) using WGS is likely to become essential in combatting AMR. For example, associations between resistance to the third generation cephalosporin, cefixime and possession of *penA* mosaic alleles have been identified in several WGS studies undertaken in *N. gonorrhoeae*.^6^, ^13^, ^14^ The PubMLST.org/neisseria website archives and annotates, at the time of writing, >7000 WGS data from multiple *Neisseria* species including *N. gonorrhoeae*.^15^ WGS data deposited in the database are annotated, gene-by-gene, enabling rapid extraction of strain information and enhancing surveillance.^16^ Pivotal to surveillance is the capacity for AMR detection to be comparable across datasets and requires AMR determinants to be annotated in a readily accessible and reproducible format available to the entire community. In this study, a catalogue of all known genes implicated in AMR is provided with genomic comparison of WGS data from a representative *N. gonorrhoeae* dataset identifying distinct gonococcal populations clustering by AMR genotype indicative of the presence of additional genomic elements associated with AMR. This included a type 4 secretion system (T4SS) also known as the gonococcal genetic island (GGI) which is known to enhance HGT through the secretion of single stranded DNA.^17^ Data presented here reveal that the T4SS was significantly associated with gonococci exhibiting reduced susceptibility to multiple antimicrobial compounds. The presence of the T4SS may therefore not only offer selective advantages to host bacteria but may also facilitate the spread of AMR in gonococcal populations.

## Materials and methods

### Isolate collections and WGS analyses

WGS data from published isolate collections included: i) 236 isolates collected from sentinel public STD clinics by the US Centers for Disease Control and Prevention Gonococcal Isolate Surveillance Project; and ii) 53 isolates of diverse origin dating from the 1980s to 2011. Isolates had been analysed for antimicrobial minimum inhibitory concentrations (MICs) to several antibiotics.^13^, ^14^ Short reads were obtained from the European Nucleotide Archive (ENA) and assembled *de novo* using VELVET in combination with VELVETOPTIMISER as previously described.^18^ The resulting contigs were uploaded to the Bacterial Isolate Genome Sequence (BIGSdb) genomics platform hosted on www.pubmlst.org/neisseria.^15^

WGS data were compared using the genome comparator tool, implemented within the PubMLST.org/neisseria website which runs the BIGSdb genomics platform.^15^, ^18^, ^19^ Using this tool, loci defined in the database or an annotated reference genome can be compared among genomes. Using a reference genome, the coding sequences within the reference annotation are extracted and compared against assembled WGS contigs. Unique allele sequences at each locus are designated with an integer starting at 1 (representing identity with the reference sequence) eventually leading to a genome-wide multi locus profile (wgMLST) from which a distance matrix can be generated and resolved into networks using the NeighborNet algorithm implemented in Splitstree.^20^ In this study, the reference genomes from *N. gonorrhoeae* isolates FA1090 (accession number NC_002946) and MS11 (accession number NC_022240) were employed.

The GGI characterised in *N. gonorrhoeae* isolate MS11 (Accession number AY803022) was used as a reference. It is composed of 62 open reading frames and, sequences from each of these were defined in the database (Supplementary Table 1).^17^ WGS were then annotated for the presence or absence of this element.

### Annotation of AMR loci

Loci defined in pubmlst.org/neisseria are allocated a value-free nomenclature using the prefix NEIS followed by 4 digits.^18^ AMR loci were designated accordingly and were linked with any number of aliases including locus tags from finished genomes or gene names (Table 1). For example, penicillin binding protein 2 was defined as NEIS1753 and was associated with the locus tag NGO1542 (from the reference *N. gonorrhoeae* isolate FA1090) and the gene name, *penA*. As alterations in promoter regions located upstream of specific loci have been found to increase antibiotic resistance,^9^ specific loci were assigned the *pro* suffix (for *pro*moter) followed by the corresponding locus prefix for the adjacent gene to differentiate them from coding sequences, e.g. ^pro^NEIS1635 (Table 1).

The plasmid containing the *tetM* gene, conferring resistance to tetracycline from *N. gonorrhoeae* 5289 (GU479466),^12^ was used to define loci NEIS2202-NEIS2249, with NEIS2210 designating the *tetM* gene. Sequences from the beta-lactamase plasmid conferring resistance to beta-lactams were retrieved from plasmid pSJ5.2 containing *bla*_*TEM1*_, and defined as NEIS2357–NEIS2360 (DQ355980) with NEIS2357 designating the *bla*_*TEM*_ gene (Table 1).^21^

The BIGSdb software includes ‘autotagger’ and “autodefiner” tools which scan deposited WGS against defined loci identifying alleles greater than or equal to 98% sequence identity. This process runs in the background and automatically updates isolate records with specific allele numbers, marking regions on assembled contiguous sequences (contigs) for any of the defined loci. Loci with sequence identity <98% were manually checked and curated. Using the molecular evolutionary analysis software MEGA v6, deduced amino acid sequences were aligned identifying polymorphic sites associated with antimicrobial resistance and enabling alleles containing these mutations to be detected (Table 2).^22^ Four copies of 16S rRNA and 23S rRNA are present in *N. gonorrhoeae* genomes. Reference sequences containing 16S and 23S rRNA along with flanking loci were created against which short reads from isolates were mapped using the Burrows–Wheeler Alignment (BWA) software package and subsequently viewed using Tablet.^23^, ^24^ Mapped reads were then visually inspected and nucleotide substitutions verified.

### Antimicrobial resistance phenotype

MIC cut-offs, guided by the US GISP antimicrobial susceptibility criteria, were defined for each antimicrobial compound (Supplementary Table 2). Phenotypic testing of AMR is the current preferred method for determining antimicrobial susceptibility and is the “gold standard” with any new approaches, such as genotypic AMR, requiring validation against this using sensitivity, specificity and predictive values. These were calculated as described previously.^25^

## Results

### *N. gonorrhoeae* wgMLST

**Figure 1:**
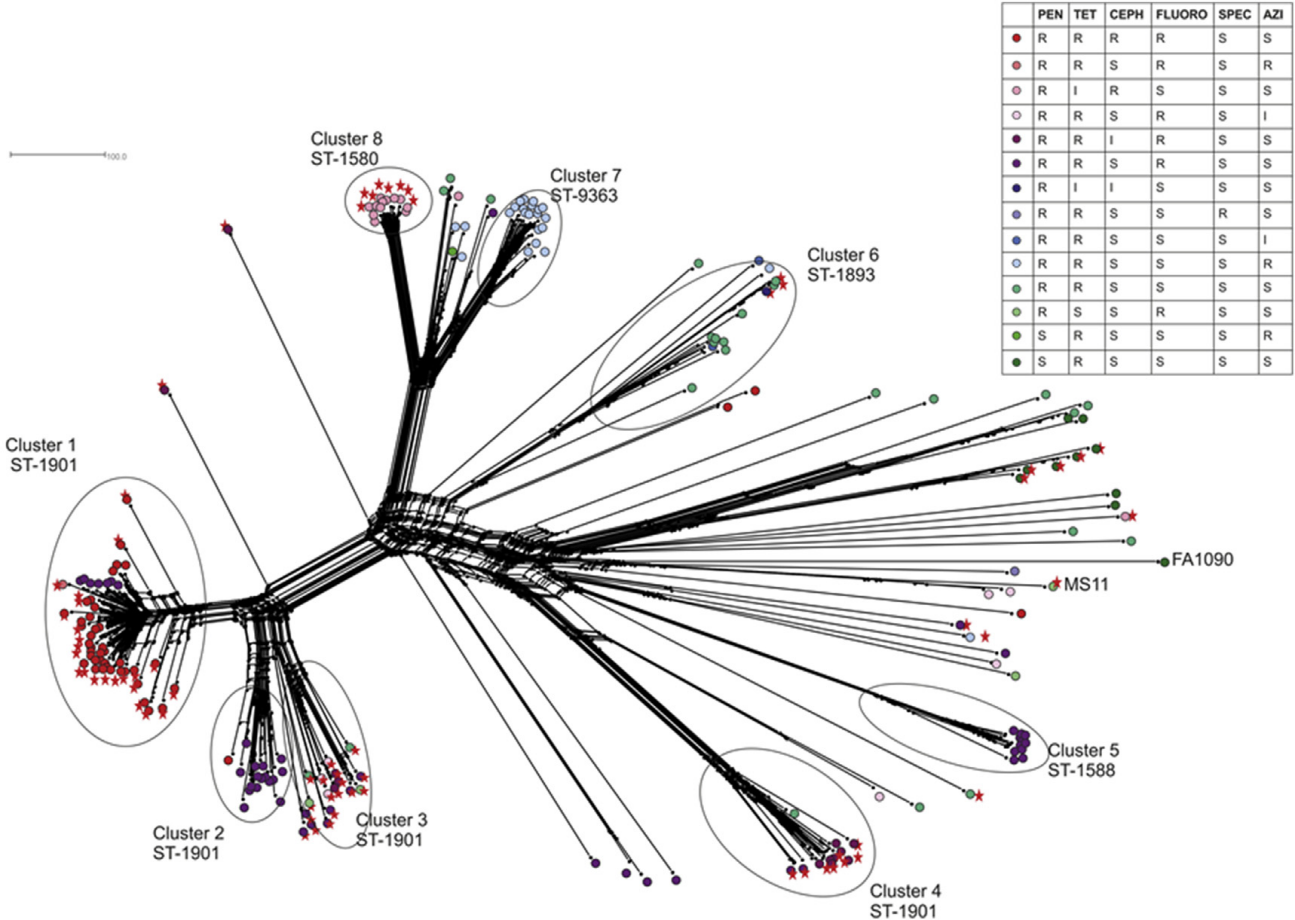
**Whole genome genealogy of *N. gonorrhoeae* isolates investigated in this study**. A neighbourNet graph depicting whole genome MLST (wgMLST) comparison of WGS data from 289 isolates. Each branch tip represents one isolate with circles colour-labelled according to AMR genotype starting with red circles depicting isolates with resistant AMR genotypes through to green circles depicting susceptible isolates. Red stars indicate the presence of the gonococcal genetic island. PEN: penicillin; TET: tetracycline; CEPH: cephalosporins; FLUORO: fluoroquinolones; SPEC: spectinomycin; AZI: azithromycin. R: resistant; S: susceptible; I: intermediate.

Whole genome analysis identified a star burst phylogeny with isolates forming discrete clusters associated with distinct AMR genotypes and the presence/absence of the T4SS, known as the gonococcal genetic island (GGI) (Fig. 1, Supplementary Table 1). A significant association between possession of the GGI and AMR to multiple compounds was identified (Supplementary Table 3). Four MLST ST-1901 clusters were apparent: Cluster 1 associated with *penA* (NEIS1753) allele 266 and isolates exhibiting resistance to multiple antimicrobial compounds as previously identified by Grad et al.; this cluster also contained the GGI^13^; Clusters 2 and 3 also included ST-1901 isolates with divergent AMR profiles compared with cluster 1, with cluster 3 including isolates with the GGI and cluster 2 without the GGI. Cluster 4 contained another group of ST-1901 isolates. Cluster 1 included several ST-1901 isolates susceptible to cephalosporins. These isolates contained *penA* (NEIS1753) allele 289 (*penA* motif XXXVIII, Table 2) which is not associated with reduced susceptibility to cephalosporins.^13^ Allelic profiles for the other AMR genes were, however, the same as the other ST-1901 isolates in this cluster.

Another group of isolates, previously identified as cluster 2 by Grad et al. but indicated here as cluster 8, were ST-1580, contained NEIS1753 allele 266 as well as the GGI but were susceptible to ciprofloxacin.^13^ These isolates possessed the smaller transferrin binding protein B gene (isotype I) implicated in iron acquisition and predominantly associated with *Neisseria meningitidis* isolates from clonal complex ST-11 as well as commensal *Neisseria*.^13^, ^26^ Cluster 7 contained isolates from ST-9363 and lacked the GGI but were resistant to azithromycin (Fig. 1). Clusters 5 and 6 included ST-1588 and ST-1893 isolates on longer branches indicative of diversity. FA1090 and MS11 were part of a large diverse group of isolates, some of which dating from the 1980s.^14^, ^27^

### AMR analysis

A catalogue of all AMR determinants in gonococci is described (Table 1). Alleles containing mutations associated with resistance were identified and linked with annotations describing principal mutations (Table 2). Mutations in *ponA* (NEIS0414) associated with resistance to beta-lactam compounds were identified in 203/289 (70%) isolates with allele 13 the most predominant (187/203, 92%) (Table 2, Supplementary Table 4). Penicillin binding protein 2, *penA* (NEIS1753) alleles 266 and 281 contained *penA* mosaic motif XXXIV, which is associated with reduced susceptibility to third generation cephalosporins; however, *penA* allele 281 contained an additional non-synonymous mutation (D101 → E) found in one isolate only, GCGS126. This isolate had a cefixime MIC >0.125 μg/ml but did not possess mutations conferring resistance in any other AMR-associated loci (Supplementary Table 4). *penA* (NEIS1753) allele 266 was found in 122/289 (42%) isolates; however, 26/122 (21%) did not have mutations associated with resistance in other AMR loci. Although these isolates exhibited reduced susceptibility to cefixime, they did not have resistant phenotypic MIC values to any of the other antimicrobials (Supplementary Table 4).

Most isolates, 282/289 (98%) contained the *porB1b* (NEIS2020) allele associated with decreased susceptibility to beta-lactams and tetracycline with AMR conferred through non-synonymous substitutions in loop III of PorB.^10^ A total of 31 distinct loop III regions were identified with those containing G120 → K and A121 → D/N mutations associated with resistant MIC values to penicillin and tetracycline (Supplementary Table 5). Only 3/289 (1%) isolates contained amino acid substitution D526 → N found in *pilQ* (NEIS0408) associated with decreased susceptibility to cefixime and ceftriaxone,^28^ however, these isolates lacked mosaic *penA* (NEIS1753), *mtrR* (NEIS1635) and *porB1b* (NEIS2020) mutations and therefore were susceptible to these compounds. Amino acid mutation S341 → N in *pilQ* (NEIS0408, also known as *pilQ* allele VII), not associated with increased resistance to cephalosporins, was found in 275/289 (95%) isolates.^28^

Plasmid mediated AMR was not prevalent with 21/289 (7%) isolates containing the beta-lactamase plasmid and 19/289 (6%) the TetM conjugative plasmid. Divergent *bla*_*TEM*_ genes have been described with *bla*_TEM1_ the most commonly found followed by *bla*_TEM135_.^11^ NEIS2357 (*bla*_*TEM*_) alleles 3 and 9 were *bla*_TEM1_ and were found in 15/21 (71%) isolates while allele 2 designated *bla*_TEM135_ and was found in 6/21 (29%) isolates. There were two NEIS2210 (*tetM*) alleles with allele 1 found in 7/19 (37%) isolates and allele 2 in 12/19 (63%) isolates (Table 2).

Most isolates, 176/289 (61%), were found with mutations S91 → F and D95 → G in *gyrA* (NEIS1320) conferring resistance to fluoroquinolones, with allele 14 the most predominant (138/176, 78%). In *parC* (NEIS1525), 184/289 (64%) isolates contained amino acid substitutions at residue 87 (S87 → R) only, with allele 104 the most predominant (145/184, 79%) and found in association with *gyrA* (NEIS1320) allele 14. All of these isolates were resistant to ciprofloxacin (Table 2). None of the isolates were found with the G410 → V substitution in *parE* (NEIS1600).^29^

Of the previously reported mutations associated with macrolide resistance, mutation C24 → P identified in *rpsE* ribosomal protein S5 (NEIS0149) was not found.^7^, ^30^, ^31^ Mutation C2599 → T in 23S rRNA was found in 25/289 (9%) isolates and these had azithromycin MIC values ≥8 μg/ml. One isolate, MUNG19, had mutation A → 2143G, 23S rRNA allele 456, and had resistant MIC to azithromycin, ≥256 μg/ml (Table 2, Supplementary Table 3). Spectinomycin resistance is conferred through deletion of codon 27 and, subsequent L28 → E substitution in *rpsE* (NEIS0149) allele 83 or mutation C1186 → T in 16S rRNA allele 1538.^7^, ^30^ Two isolates, ATL0121 and MUNG18, were found with either of these mutations, however, spectinomycin phenotypic values were unavailable (Table 2, Supplementary Table 2).

The adenine deletion in the 13bp promoter region associated with increased expression of the MtrCDE efflux pump was found in 178/289 (62%) isolates (^pro^NEIS1635 allele 3)^9^ and was associated with mutations in many of the other AMR loci including *penA* (NEIS1753), *ponA* (NEIS0414) and *porB* (NEIS2020). Four isolates were found with an A → C substitution in the promoter region, ^pro^NEIS1635 allele 4, with these isolates also containing premature stop codons in *mtrR* gene consistent with putative non-functional MtrR proteins. These were associated with resistant MIC to azithromycin^8^ (Table 2). Mutations associated with high AMR MIC values were not detected in the efflux pumps MacAB and FarAB.^32^, ^33^ None of the isolates were found with nucleotide substitution G → T in the −10 promoter region (5′-TA**G**AAT-3′) upstream of *macA* (^pro^NEIS0488) and no significant mutations were found in the transcriptional regulator, NEIS0374 (*farR*).^34^ Overexpression of NEIS0763 (*norM*) may occur when a T → C nucleotide occurs in the −35 box in the promoter region (^pro^NEIS0763) (TTGACG to **C**TGACG)^35^ and all of the isolates contained this substitution.

### Phenotype vs genotype correlation

High congruence was observed between phenotypic AMR and the predicted genotypic AMR (Table 3). Discrepancies occurred when comparing beta-lactam resistance profiles with, for example, nine isolates containing MIC values ≤1 μg/ml to penicillin but which had AMR amino acid mutations associated with resistance in loci NEIS1753, NEIS0414, NEIS2020, NEIS0408 and ^pro^NEIS1635 for which other isolates with the same mutations had MIC values ranging from 2 to 8 μg/ml to penicillin. In addition, four isolates with an AMR genotype had reduced susceptibility to cefixime and penicillin but were susceptible to ceftriaxone and cefpodoxime. Two isolates had genotypic profiles consistent with reduced susceptibility to cefixime but did not have a corresponding resistant phenotype. Three isolates contained a beta-lactamase plasmid but had MICs ≤1 to penicillin.

PPV scores were over 95% for each antimicrobial compound consistent with genotypic AMR performing as well as phenotypic AMR in detecting antimicrobial resistance (Table 4). NPV scores indicated whether isolates with a susceptible phenotype also had a susceptible genotype and NPV scores were low for penicillin and tetracycline but high for cefixime, ciprofloxacin and azithromycin (Table 4). Sensitivity and specificity scores were high for all compounds.

## Discussion

Direct deduction of resistance from WGS data provides an important opportunity for the enhanced surveillance of AMR for public health benefit. Gonococcal AMR is, however, a complex phenotype resulting from single to multiple genetic changes often occurring in synergy and resulting in increasing levels of antimicrobial resistance to several compounds with the added uncertainty that additional unknown genetic elements may also be playing a role.^3^, ^5^ The complexity of gonococcal AMR is further exacerbated by the presence of multiple gene names and lack of web-accessible repositories with which sequence data can be queried. In this study, all of the known genes implicated in AMR were catalogued defining AMR determinants in a readily accessible, reproducible format found on the www.pubmlst.org/neisseria website, which hosts WGS data from multiple *Neisseria* species (Table 1, Table 2).^19^

Gene-by-gene annotation of AMR loci, combined with wgMLST analysis, identified clusters of isolates with distinct AMR genotypes. Some of these also possessed the GGI, a T4SS known to facilitate HGT through the secretion of single stranded DNA (ssDNA) into the extracellular environment (Fig. 1, Supplementary Table 1).^17^ T4SSs are mobile genetic elements and play a major role in HGT allowing bacteria to outcompete other bacterial species through the acquisition of a variety of fitness genes including catabolic, virulence and antibiotic resistance. For example, antimicrobial resistance in *Haemophilus influenzae* has been shown to be associated with the acquisition of integrative conjugative elements known as ICEs, a type of T4SS.^36^ It is also known that mobile genetic elements such as plasmids, phages and genomic islands play an important role in the emergence of pathogenic Enterobacteriaceae.^37^ A number of hypothetical genes remain to be characterised in the GGI which may offer additional selective advantages to host gonococci (Supplementary Table 1). The association, however, of the T4SS in this study with *N. gonorrhoeae* isolates exhibiting reduced susceptibility to multiple antimicrobial compounds is consistent with the likelihood that this element will accelerate the spread of AMR.

Expansion of distinct gonococcal populations may also be promoted through the activity of toxin–antitoxin (TA) subunits encoded by the genes, *ydhB* (NEIS2281) and *ydcA* (NEIS2282), located in the GGI (Supplementary Table 1). TA are common features of mobile genetic elements and the negative effects of cell growth conferred by the toxin are suppressed by an antitoxin. Cells lacking the mobile genetic element and, therefore the TA, are harmed by the toxin producing cells, which are themselves immune due to possession of the antitoxin.^38^, ^39^ Thus, the presence of the GGI may have been a significant factor in the expansion in the Western Hemisphere of gonococci belonging to ST-1901 (NG-MAST ST-1407). In addition, isolates possessing both the GGI and plasmid mediated AMR were not prevalent in this dataset. Many of GGI encoded genes show similarity to those from the *Escherichia coli* F-plasmid conjugation system and, the order of the genes in the GGI is highly similar to the IncF family of conjugative plasmids, consistent with the GGI being an ancestral chromosomally inserted plasmid.^17^, ^40^ In particular, the DNA methylases, *ydg* (NEIS2288) and *ydhA* (NEIS2289) can be found which may enable within host competition between plasmids consistent with the low prevalence of isolates here possessing both a plasmid and the GGI^41^ (Supplementary Tables 1 and 4).

The increasing number of bacteria becoming resistant to multiple antimicrobials is a major global concern with health officials warning of the possibility of untreatable bacterial infections.^42^ The tools developed in this study present a means through which AMR can be deduced from WGS while also permitting AMR genotypes to be compared between isolates and linked with additional genomic data. Furthermore, the availability of a web-accessible database enables globally distinct isolate collections, where selection pressures will be different, to be compared, thereby enriching surveillance. Concordance was high between phenotypic and genotypic AMR with most of the discrepancies observed for the beta-lactam compounds and tetracycline, for which multiple genetic components are implicated in conferring resistance (Table 3, Table 4). In most cases, AMR genotypes were identified which did not correlate with AMR phenotypes (*i.e.* isolates had susceptible phenotypes despite the presence of resistant genotypes). These correlated with some of the lower NPV scores obtained for penicillin and tetracycline (Table 4). The high PPV, specificity and sensitivity values are encouraging, however, and indicate that molecular AMR diagnosis may be useful in surveillance particularly in settings where diagnosis relies on nucleic amplification tests (NAATS) and cultures are not available (Table 4).^43^

*N. gonorrhoeae* has developed resistance to all antimicrobials recommended in the first-line empirical treatment of gonorrhoeae and in order to understand and limit the onset of an era of untreatable gonorrhoea, it is essential that factors underpinning the acquisition of antimicrobial resistance are understood and monitored. The data and tools presented here provide a model in which this can be accomplished using an easily accessible database with the likelihood that such interfaces will become particularly important as more WGS data become available.

## Funding

This study was jointly funded by a Wellcome Institutional Strategic Support Fund (WTISSF) and the Oxford Martin School, University of Oxford (H2RXJo00). MCJM was supported by the Wellcome Trust (087622). YHG was supported by the National Institutes of Health (K08-AI104767-01). DLT supported by the CDC and CDC's Office of Advanced Molecular Detection (AMD-18).

## Conflict of interest

The authors declare no competing interests.

## Figures and Tables

**Table 1:** Antimicrobial resistance loci defined in pubMLST.org/neisseria.

Locus	Gene (aliases)	Product
**Beta-lactams**		
NEIS0408	*pilQ/penC* (NGO0094)	Type IV pilus biogenesis protein
NEIS0414	*ponA* (NGO0099; NMB1807)	Penicillin binding protein 1; peptidoglycan glycosyltransferase (EC2.4.1.129)
NEIS1753	*penA* (NGO1542; NMB0413)	Penicillin binding protein 2; peptidoglycan glycosyltransferase (EC2.4.1.129)
NEIS2020	*porB/penB* (NGO1812; NMB2039)	Major outer membrane porin
**Fluoroquinolones**		
NEIS1320	*gyrA* (NGO0629; NMB1384	DNA gyrase subunit A (EC5.99.1.3)
NEIS1525	*parC* (NGO1259; NMB1605)	DNA topoisomerase IV subunit A
NEIS1600	*parE* (NGO1333; NMB1682)	DNA topoisomerase IV subunit B (EC5.99.1)
**Macrolides and aminoglycosides**		
16S rRNA		16S rRNA
NEIS0149	*rpsE*	30S ribosomal protein S5
23S rRNA		23S rRNA
**Efflux pumps**		
NEIS0488	*macA* (NGO1440)	Macrolide-specific efflux pump protein; ABC transporter
NEIS0489	*macB* (NGO1439)	Macrolide-specific efflux pump protein; ABC transporter
^*pro*^NEIS0488	*macAB* promoter region	Intergenic promoter region (73bp upstream of NEIS0488)
NEIS1635	*mtrR* (NGO1366; NMB1717)	Efflux pump transcriptional regulator repressor
^*pro*^NEIS1635	*mtrR* promoter region	Intergenic promoter region (67nt upstream of *mtrR*)
NEIS1852	*farB* (NGO1682)	Efflux pump protein, fatty acid resistance
NEIS1853	*farA* (NGO1683)	Efflux pump protein, fatty acid resistance; homopolymeric tract
NEIS0374	*farR*/*marR* (NGO0058)	MarR family transcriptional regulator
NEIS0763	*norM* (NGO0395)	Multidrug and toxin extrusion (MATE) family efflux pump
^*pro*^NEIS0763	*norM* promoter region	Intergenic promoter region (104bp upstream of NEIS0763)
**Plasmids**		
NEIS2357-2360 NEIS2357: *bla*_*TEM*_	pTem plasmid	Beta-lactamase encoded plasmid
NEIS2202-2249 NEIS2210: *tetM*	TetM plasmid	Tetracycline resistant plasmid

**Table 2:** Antimicrobial resistance alleles containing mutations known to confer resistance.

Locus	Known amino acid substitutions associated with resistance	Principal alleles with mutations conferring AMR resistance (MIC values where available)
**Beta-lactams**		
NEIS0408 (*pilQ/penC*)	QAATPAKQ insertion at 180 D526 → N *pilQ* allele I Q172 → E *pilQ* allele II N648 → S *pilQ* allele III N432 → S; N648 → S *pilQ* allele IV S341 → N; D494 → N; N648 → S *pilQ* allele V S341 → N; N648 → S *pilQ* allele VI S341 → N *pilQ* allele VII S341 → N; G500 → S *pilQ* allele VIII AKQQAAAP deletion at 147; S341 → N *pilQ* allele IX	Alleles 332, 575, and 598: *pilQ* I Alleles 184, 659, 662 and 667: *pilQ* III Allele 602: *pilQ* IV Alleles 316, 317, and 664: *pilQ* V Alleles 23,318, 319, 322 and 327: *pilQ* VI Alleles 22, 251, 314, 320, 321, 323, 324, 325, 326, 328, 329, 330, 331, 590, 660, 661, 668, and 666: *pilQ* VII
NEIS0414 (*ponA*)	L421 → P	Allele 13 (PEN 0.25–16; TET: 0.25–64*; CFX: 0.008–1; CEF: 0.008–0.25; CPDF: 0.015–4) Allele 48 (PEN 1–4; TET: 0.5–32*; CFX: 0.008–0.06; CEF: 0.008–0.06; CPDF: 0.03–1) Allele 222 (PEN 0.5; TET: 0.5; CFX: 0.015; CEF: 0.008; CPDF: 0.03) Allele 224 (PEN 4; CFX: 0.03; TET: 2; CEF: 0.03; CPDF: 0.125) Allele 225 (PEN 2–4; TET: 2; CFX: 0.03–0.06; CEF: 0.03–0.06; CPDF: 0.125)
NEIS1753 (*penA*)	I312 → M, V316 → T, D345 → a, A501 → V/P, F504 → L, N512 → Y, G545 → S, P551 → S/L	Alleles 14 and 511: *penA* motif X (TET: 1–2; CFX: 0.5) Alleles 286, 291, 292 and 517: *penA* motif VII (PEN 1–16*; TET: 0.5–16; CFX 0.03–0.06; CEF: 0.03–0.06; CPDF: 0.125–0.25) Alleles 266, 281, 498, and 547: *penA* motif XXXIV (PEN 0.25–8; TET: 0.25–16*; CFX 0.015–0.5; CEF: 0.008–0.25; CPDF: 0.015–4) Allele 500: *penA* motif XXXIV with A501 → P amino acid substitution (TET: 2; CFX: 1) Alleles 289: *penA* motif XXXVIII (PEN: 0.25–1; TET: 0.5–2; CFX 0.015–0.03; CEF: 0.008–0.015; CPDF: 0.06–0.125)
NEIS2020 (*porB*)	G120 → K, A121 → D Novel mutations identified in this study: G120 → D/N/R, A121 → G/N/S	Alleles: 512, 517, 521, 523, 524, 526, 528, 530, 531, 534, 539, 540, 541, 542, 544, 545, 546, 547, 548, 550, 551, 552, 553, 554, 556, 557, 558, 560, 561, 562, 564, 565, 566, 568, 569, 570, 571, 573, 574, 575, 576, 577, 578, 579, 580, 581, 583, 584, 585, 586, 587, 588, 589, 590, 593, 594, 628, 629, 631, 632, 633, 634, 635, 636, 637, 638, 639, 647, 671, 728, 729, 786, 810, 877, 882, 968, 969, 970, 971, 974, 975, 976, 977, 982, 983, 985, 987, 988, 989, 990 (PEN: 0.25–16*; CFX: 0.004–1; CEF: 0.008–0.25; CPDF: 0.015–4)
**Fluoroquinolones**		
NEIS1320 (*gyrA*)	S91 → F, D95 → G/A/N/Y	Allele 14 (CIP: 0.015–32), allele 193 (CIP: 0.015–32), allele 231 (CIP: 4–32), allele 233 (CIP: 2–8), allele 234 (CIP: 1–16), allele 236 (CIP: 1), allele 237 (CIP: 16), allele 239 (CIP: 32), allele 409 (CIP: 1–16)
NEIS1525 (*parC*)	D86 → N, S87 → I/N/R, S88 → P	Allele 104 (CIP: 0.015–32), allele 243 (CIP: 2–32), allele 246 (CIP: 1–2), allele 252 (CIP: 16), allele 253 (CIP: 16), allele 255 (CIP: 4), allele 257 (CIP: 32), allele 258 (CIP: 8), allele 488 (CIP: 0.015–32), allele 508 (CIP: 0.015–32)
NEIS1600 (*parE*)	G410 → V	None of the isolates were found with this substitution
**Macrolides and aminoglycosides**		
16S rRNA	C1192 → T (*Escherichia coli* numbering; this corresponds to 1186 in *N. gonorrhoeae*)	Allele 1538
NEIS0149 (*rpsE*)	Deletion of codon 27 K28 → E; T24 → P	Allele 83
23S rRNA	C2599 → T; A2143 → G	Allele 431 (AZI: 2–16) Allele 432 (AZI: 16) Allele 436 (AZI: 2) Allele 439 (AZI: 2) Allele 456 (AZI: >256)
**Efflux pumps**		
NEIS0488 (*macA*)	No mutations described	No mutations identified associated with AMR
NEIS0489 (*macB*)	No mutations described	No mutations identified associated with AMR
^pro^NEIS0488 *macAB* promoter region	G → T substitution in −10 promoter region (5′-TA**G**AAT-3′) increases transcription	None of the isolates had this substitution
NEIS1635 (*mtrR*)	Premature stop codons	Allele 367 (PEN: 0.25–0.5; TET: 0.5–1; CFX: 0.015–0.03; CEF: 0.008; CPDF: 0.015–0.03; AZI: 8–16) Allele 368 (PEN: 1–8; TET: 16–64*; CFX: 0.015–0.03; CEF: 0.008–0.03; CPDF: 0.03–0.06; AZI: 0.03–0.25) Allele 370 (PEN: 2; TET: 2; CFX: 0.06; CEF: 0.06; CPDF: 0.125; AZI: 0.25) Allele 371 (PEN: 1; TET: 0.5–16*; CFX: 0.015; CEF: 0.008; CPDF: 0.015; AZI: 0.06–0.5) Allele 373 (n/a) Allele 376 (n/a)
^*pro*^NEIS1635 (*mtrR* promoter region)	Adenosine deletion in efflux pump MtrR promoter region	^*pro*^NEIS1635 allele 1 (PEN: n/a; TET: 4; CFX: 0.004; AZI: 0.125) ^*pro*^NEIS1635 allele 2 (PEN: 2; TET: 1; CFX: 0.03; CEF: 0.015; CPDF: 0.06; CIP: 16; AZI: 0.5) ^*pro*^NEIS1635 allele 3 (PEN: 0.25–16; TET: 0.25–32*; CFX: 0.015–1; CEF: 0.008–0.25; CPDF: 0.015–4; CIP: 0.015–32; AZI: 0.03–2) ^*pro*^NEIS1635 allele 4 (PEN: 0.25–0.5; TET: 0.5–1; CFX: 0.015–0.03; CEF: 0.008; CPDF: 0.015–0.03; CIP: 0.015; AZI: 8–16) ^*pro*^NEIS1635 allele 5 (PEN: n/a; TET: 0.5; CFX: 0.016; AZI: 2)
NEIS1852 (*farB*)	No mutations described	n/a
NEIS1853 (*farA*)	No mutations described	n/a
NEIS0374 (*farR/marR*)	Regulated by MtrR such that over expression of MtrR results in decreased expression of FarAB	n/a
NEIS0763 (*norM*)	Promoter region	n/a
^pro^NEIS0763 *norM* promoter region	CTGACG instead of TTGACG substitution in the −35 box resulting in overexpression of NorM	n/a
**Plasmids**		
NEIS2357 (*bla-TEM*)		Allele 3 and allele 9: *bla*_*TEM*_*1* Allele 2: *bla*_*TEM*_*135*
NEIS2210 (*tetM*)		Alleles 2, 3, and 9

**Table 3:** AMR Phenotype and genotype concordance in isolates from the US-GISP study.

Antimicrobial	Number of isolates in the US-GISP study with concordant phenotype and genotype
Penicillin	205/236 (87%)
Tetracycline	216/236 (92%)
Cephalosporins	190/236 (81%)
Ciprofloxacin	234/236 (99%)
Azithromycin	232/236 (98%)
Spectinomycin	236/236 (100%)

**Table 4:** Sensitivity and Specificity of genotype vs phenotype calculated for the US-GISP isolates.

Antimicrobial	Sensitivity (%)	Specificity (%)	Positive predictive value (PPV) (%)	Negative predictive value (NPV) (%)
Penicillin	80.59	96.61	98.56	63.33
Tetracycline	98.68	100	100	71.43
Cefixime	98.37	99.12	99.18	98.25
Ciprofloxacin	98.82	100	100	97.10
Azithromycin	100	98.04	88.89	100
Spectinomycin	nd	nd	nd	nd

PPV was calculated as the proportion of isolates with a resistant AMR genotype to have a resistant AMR phenotype: PPV = a/a + b where a (true positive)/a + b (true positive + false positive). NPV calculated the proportion of isolates with a susceptible AMR genotype to also have a susceptible phenotype: NPV = d/c + d where d (true negative)/c + d (false negative + true negative). Sensitivity calculated how likely a resistant AMR genotype was able to detect an isolate with a resistant AMR phenotype: a/a + c where a (true positive)/a + c (true positive + false negative). Specificity identified how likely a susceptible AMR genotype was able to detect an isolate with a susceptible AMR phenotype: d/b + d where d (true negative)/b + d (true negative + false positive).
