# Supplementary material for: Genomic analyses of Neisseria gonorrhoeae reveal an association of the gonococcal genetic island with antimicrobial resistance
Source: J Infect. 2016 Dec;73(6):578–87. doi: 10.1016/j.jinf.2016.08.010 (PMC5127880; doi:10.1016/j.jinf.2016.08.010)
Supplement: Supplementary file 1 [file mmc1.docx]

**Supplementary Table 1. List of loci found in the GGI showing NEIS nomenclature, gene name and proposed function.**

| **NEIS number** | **gene name** | **product** | **gene length** |
| --- | --- | --- | --- |
| NEIS2315 | *yaa* | conjugal transfer protein | 579 |
| NEIS2250 | *traD* | putative docking protein | 2465 |
| NEIS2251 | *traI* | putative nicking enzyme | 2553 |
| NEIS2252 | *yaf* | hypothetical protein | 420 |
| NEIS2253 | *ltgx* | peptidoglycan hydrolase | 462 |
| NEIS2254 | *yag* | outer membrane protein | 483 |
| NEIS2255 | *traA* | putative transfer protein | 252 |
| NEIS2256 | *traL* | pilus assembly | 282 |
| NEIS2257 | *traE* | pilus biogenesis | 651 |
| NEIS2258 | *traK* | pilus assembly | 735 |
| NEIS2259 | *traB* | conjugal transfer | 1314 |
| NEIS2260 | *dsbC* | protein disulphide isomerase | 720 |
| NEIS2261 | *traV* | putative transfer protein | 582 |
| NEIS2262 | *traC* | pilus assembly | 2677 |
| NEIS2263 | *ybe* | hypothetical protein | 549 |
| NEIS2264 | *trbL* | conjugal transfer | 534 |
| NEIS2265 | *traW* | pilus biogenesis | 657 |
| NEIS2266* | *traU* | pilus biogenesis | 1119 |
| NEIS2267 | *trbC* | conjugal transfer | 753 |
| NEIS2268 | *ybi* | mating-pair stabilisation | 1245 |
| NEIS2269 | *traN* | mating-pair stabilisation | 1584 |
| NEIS2270 | *ycb* | hypothetical protein | 426 |
| NEIS2271 | *traF* | pilus assembly | 792 |
| NEIS2272 | *traH* | pilus assembly | 1506 |
| NEIS2273 | *traG* | pilus assembly/mating-pair stabilisation | 2919 |
| NEIS2274 | *atlA* | peptidoglycan transglycosylase | 546 |
| NEIS2275 | *ych* | hypothetical protein | 159 |
| NEIS2276 | *exp1* | exported protein | 324 |
| NEIS2311 | *eppA* | eppA | 444 |
| NEIS2312 | *ych1* | hypothetical protein | 165 |
| NEIS2277 | *cspA* | RNA/ssDNA binding protein | 579 |
| NEIS2278 | *exp2* | hypothetical protein | 351 |
| NEIS2279 | *yda* | hypothetical protein | 615 |
| NEIS2280 | *ydbA* | hypothetical protein | 984 |
| NEIS2281 | *ydhB* | putative toxin resembling yhaV | 450 |
| NEIS2282 | *ydcA* | putative antitoxin resembling mazE | 303 |
| NEIS2283 | *ydcB* | hypothetical protein | 465 |
| NEIS2284 | *ydd* | hypothetical protein | 483 |
| NEIS2285 | *ydeA* | hypothetical protein | 168 |
| NEIS2286 | *ydeB* | hypothetical protein | 516 |
| NEIS2287 | *ydf* | hypothetical protein | 582 |
| NEIS2288 | *ydg* | putative DNA methylase; type I restriction endonuclease subunit M | 342 |
| NEIS2289 | *ydhA* | class I adenine-specific DNA methylase | 1122 |
| NEIS2290 | *ydhB* | hypothetical protein | 225 |
| NEIS2291 | *ydi* | hypothetical protein | 258 |
| NEIS2292 | *yea* | putative helicase | 2484 |
| NEIS2293 | *yeb* | putative N-acetyltransferase | 492 |
| NEIS2294 | *yecA* | hypothetical protein | 285 |
| NEIS2295 | *yecB* | repeat containing protein | 342 |
| NEIS2296 | *yedA* | hypothetical protein | 582 |
| NEIS2297 | *yedb* | hypothetical protein | 516 |
| NEIS2298 | *yee* | hypothetical protein | 369 |
| NEIS2314 |  | hypothetical protein | 324 |
| NEIS2313 |  | hypothetical protein | 450 |
| NEIS2299 | *yegA* | hypothetical protein | 591 |
| NEIS2300 | *yegB* | hypothetical protein | 99 |
| NEIS2301 | *yeh* | hypothetical protein | 540 |
| NEIS2302 | *topB* | DNA topoisomerase | 2040 |
| NEIS2303 | *ssbB* | single-stranded DNA binding protein | 432 |
| NEIS2304 | *yfa* | hypothetical protein | 318 |
| NEIS2305 | *yfb* | hypothetical protein | 1050 |
| NEIS2306 | *yfd* | putative TonB-like transporter | 1356 |
| NEIS2307 | *yfeA* | hypothetical protein | 756 |
| NEIS2308 | *yfeB* | hypothetical protein | 606 |
| NEIS2309 | *parB* | chromosome partitioning | 1491 |
| NEIS2310 | *parA* | chromosome partitioning | 888 |

The GGI characterised in *N. gonorrhoeae* isolate MS11 (Accession number AY803022) was used as a reference. It is composed of 62 open reading frames and, sequences from each of these were defined as separate loci in the database ([1](#_ENREF_1)). The genes, *eppA* and *ych1*, associated with GGI found in some *Neisseria meningitidis* isolates were also included in order to identify such GGI (accession number DQ835990) ([2](#_ENREF_2), [3](#_ENREF_3)). The product of each translated sequence was confirmed using the Pfam basic local alignment search tool at pfam.xfam.org with any predicted domains of interest included in the locus record. Contigs containing the GGI were further examined using the genome annotation tool, Artemis ([4](#_ENREF_4)) enabling the identification of two additional loci, NEIS2313 and NEIS2314, resulting in a GGI island containing 64 genes.

**Supplementary Table 2. MIC cut-offs used**

| **Antimicrobial** | **MIC cut-off (µg/ml)** | **Susceptibility level above cut-off** |
| --- | --- | --- |
| Penicillin | ≥ 2 | Resistant |
| Tetracycline | ≥ 2 | Resistant |
| Ciprofloxacin | ≥ 1 | resistant |
| Cefpodoxime | ≥ 1 | reduced susceptibility |
| Azithromycin | ≥ 2 | reduced susceptibility |
| Ceftriaxone | ≥ 0.125 | elevated MIC |
| Cefixime | ≥ 0.25 | elevated MIC |

**Supplementary Table 3. Proportion of isolates with or without the GGI in association with antimicrobial resistance based on MIC profiles where available and also on molecular characterisation of antimicrobial resistance genes.**

| **Based on AMR phenotype (MIC values)** | |  |  |
| --- | --- | --- | --- |
| Antimicrobial | GG+ isolates with elevated MICs | GG- isolates with elevated MICs | P value |
| Cefixime | 56% (120/216) | 4% (3/72) | <0.0001**** |
| Tetracycline | 70% (151/216) | 54% (39/72) | 0.0146* |
| Azithromycin | 2% (5/216) | 35% (25/72) | <0.0001**** |
| **Based on AMR genotype** | |  |  |
| Penicillin | 61% (132/216) | 24% (17/72) | <0.0001**** |
| Tetracycline | 70% (151/216) | 54% (39/72) | 0.0146* |
| Ciprofloxacin | 74% (160/216) | 33% (24/72) | <0.0001**** |
| Cefpodoxime | 54% (116/216) | 0 | <0.0001**** |
| Azithromycin | 2% (5/216) | 35% (25/72) | <0.0001**** |
| Ceftriaxone | 14% (31/216) | 1% (1/72) | 0.0024** |
| Cefixime | 56% (120/216) | 4% (3/72) | <0.0001**** |

* significant; ** very significant; **** extremely significant

**Supplementary Table 4.** **Allelic variants of loci associated with antimicrobial resistance combined with isolate MIC profiles where available**

| **Isolate name; accession number** | **Beta-lactams** | | | | | | | | **Fluoroquinolones** | | | **Tetracycline** | | **Macrolides and aminoglycosides** | | |
| --- | --- | --- | --- | --- | --- | --- | --- | --- | --- | --- | --- | --- | --- | --- | --- | --- |
|  | **PEN MIC** | **CRO MIC** | **CPD MIC** | **CFM MIC** | **NEIS1753**  **(*penA);***  **NEIS2357 *(bla-TEM)*** | **NEIS0414**  **(*ponA)*** | **NEIS2020**  **(*porB)*** | **NEIS1635**  **(*mtrR);***  ***^pro^*NEIS1635 (*mtrR* promoter)** | **CIP**  **MIC** | **NEIS1320**  ***(gyrA)*** | **NEIS1525**  ***(parC)*** | **MIC** | **NEIS2210 *(tetM)*** | **AZM**  **MIC;**  **STR MIC** | **16S rRNA;**  **NEIS0149 (*rpsE)*** | **23S rRNA** |
| *GCGS001; ERR191730 | 4 | 0.125 | 2 | 0.25 | **266** | **13** | **517** | 13; **3** | 16 | **14** | **104** | 2 |  | 1; 1 | 41; 4 | 437 |
| *GCGS002; ERR191731 | 1 | 0.03 | 0.06 | 0.015 | 288 | **13** | **585** | 188; **3** | 16 | **14** | **104** | 1 |  | 1; 1 | truncated; 4 | 437 |
| *GCGS003; ERR191732 | 4 | 0.125 | 1 | 0.25 | **266** | **13** | **517** | 13; **3** | 16 | **14** | **104** | 4 |  | 1; 1 | 41; 4 | 387 |
| *GCGS004; ERR191733 | 2 | 0.03 | 0.06 | 0.015 | 288 | **13** | **576** | 188; **3** | 16 | **14** | **104** | 4 |  | 1; 1 | truncated; 4 | 437 |
| *GCGS005; ERR191734 | 4 | 0.06 | 1 | 0.5 | **266** | **13** | **551** | 13; **3** | 16 | **14** | **104** | 4 |  | 1; 1 | 41; 4 | 387 |
| *GCGS006; ERR191735 | 2 | 0.03 | 0.06 | 0.015 | 288 | **13** | 590 | 13; **3** | 8 | truncated | **258** | 2 |  | 1; 1 | 41; 4 | 448 |
| *GCGS007; ERR191736 | 2 | 0.06 | 1 | 0.25 | **266** | **13** | **565** | 13; **3** | 16 | **14** | **104** | 2 |  | 0.5; 1 | 41; 4 | 387 |
| GCGS008; ERR191737 | 0.5 | 0.008 | 0.03 | 0.015 | 228 | **13** | 533 | 191; 6 | 1 | 236 | **246** | 16 | **2** | 0.25; 1 | 40; 3 | 434 |
| GCGS009; ERR191738 | 1 | 0.03 | 0.25 | 0.25 | 357 | 8 | 523 | 12; 6 | 0.015 | 230 | 242 | 2 |  | 0.25; 1 | 41; 3 | 430 |
| GCGS010; ERR191739 | 0.25 | 0.015 | 0.03 | 0.015 | 166 | 8 | 511 | 12; 6 | 0.015 | 230 | 242 | 0.5 |  | 0.5; 1 | 41; 3 | 430 |
| *GCGS011; ERR191740 | 2 | 0.06 | 1 | 0.06 | 293 | **48** | 578 | 12; 6 | 0.015 | 232 | 244 | 4 |  | 0.25; 1 | 41; 3 | 430 |
| *GCGS012; ERR191741 | 1 | 0.06 | 0.25 | 0.06 | 286 | **48** | **562** | 12; 6 | 0.015 | 232 | 244 | 2 |  | 0.25; 1 | 41; 3 | 443 |
| *GCGS017; ERR191746 | 4 | 0.06 | 2 | 0.25 | **266** | **13** | **517** | 13; **3** | 32 | **14** | **104** | 4 |  | 1; 1 | 41; 4 | 387 |
| GCGS018; ERR191747 | 0.25 | 0.008 | 0.015 | 0.015 | 166 | 8 | 513 | 187; 6 | 0.015 | 230 | 242 | 1 |  | 8; 1 | 41; 3 | **431** |
| *GCGS019; ERR191748 | 4 | 0.06 | 2 | 0.25 | **266** | **13** | **517** | 13; **3** | 32 | **14** | **104** | 4 |  | 1; 1 | 1528; 4 | 387 |
| *GCGS020; ERR191749 | 1 | 0.008 | 0.03 | 0.015 | 23 | **13** | 572 | 13; **3** | 2 | **14** | **104** | 2 |  | 0.5; 1 | 41; 4 | 10 |
| *GCGS021; ERR191750 | 4 | 0.06 | 2 | 0.25 | **266** | **13** | **517** | 13; **3** | 32 | **14** | **104** | 4 |  | 1; 1 | 1528; 4 | 387 |
| *GCGS022; ERR191751 | 4 | 0.06 | 0.125 | 0.06 | 21 | **13** | **544** | 13; **3** | 16 | **231** | **243** | 4 |  | 0.5; 1 | 1530; 3 | 231 |
| *GCGS023; ERR191752 | 2 | 0.03 | 0.5 | 0.25 | **266** | **13** | **547** | 13; **3** | 16 | **14** | **104** | 4 |  | 0.5; 1 | 40; 4 | 387 |
| GCGS024; ERR191753 | 16 | 0.06 | 0.125 | 0.06 | 292;  **3** | **13** | **547** | 13; **3** | 4 | truncated | 256 | 16 | **1** | 0.5; 1 | 41; 3 | 430 |
| *GCGS025; ERR191754 | 2 | 0.06 | 1 | 0.25 | **266** | **13** | **517** | 13; **3** | 16 | **14** | **104** | 2 |  | 0.5; 1 | 41; 4 | 387 |
| *GCGS026; ERR191755 | 8 | 0.06 | 0.25 | 0.06 | 21 | **13** | **583** | 13; **3** | 16 | **231** | **104** | 1 |  | 0.5; 1 | 40; 3 | 231 |
| *GCGS027; ERR191756 | 2 | 0.03 | 1 | 0.25 | **266** | **13** | **517** | 13; **3** | 32 | **14** | **104** | 4 |  | 0.5; 1 | 41; 4 | 387 |
| *GCGS028; ERR191757 | 0.5 | 0.008 | 0.06 | 0.015 | 289 | **13** | **552** | 13; **3** | 8 | **14** | **104** | 2 |  | 1; 1 | 1528; 4 | 387 |
| *GCGS029; ERR191758 | 2 | 0.06 | 1 | 0.25 | **266** | **13** | **517** | 13; **3** | 32 | **14** | **104** | 4 |  | 0.5; 1 | 1528; 4 | 387 |
| *GCGS030; ERR191759 | 0.5 | 0.008 | 0.06 | 0.015 | **289** | **13** | **553** | 13; **3** | 8 | **14** | **104** | 2 |  | 1; 1 | 41; 4 | 387 |
| *GCGS031; ERR191760 | 2 | 0.03 | 2 | 0.5 | **266** | **13** | **517** | 13; **3** | 32 | **14** | **104** | 4 |  | 0.5; 1 | 41; 4 | 387 |
| GCGS032; ERR191761 | 4 | 0.03 | 0.125 | 0.03 | 283 | **13** | **524** | 13; **3** | 16 | **231** | **243** | 2 |  | 0.5; 1 | 40; 3 | 231 |
| *GCGS033; ERR191762 | 4 | 0.06 | 1 | 0.25 | **266** | **13** | **593** | 13; **3** | 16 | **14** | **104** | 0.5 | TetM -ve plasmid | 0.25; 1 | 41; 4 | 437 |
| *GCGS034; ERR191763 | 2 | 0.06 | 0.125 | 0.06 | 287 | **13** | **589** | 18; **3** | 32 | **239** | **257** | 2 |  | 1; 1 | 40; 3 | 447 |
| GCGS035; ERR191764 | 4 | 0.125 | 2 | 0.25 | **266** | **13** | **557** | 13; **3** | 32 | **14** | **104** | 4 |  | 1; 1 | 41; 4 | 437 |
| *GCGS036; ERR191765 | 2 | 0.015 | 0.06 | 0.03 | 288 | **13** | **584** | 194; 2 | 16 | **14** | **104** | 4 |  | 0.5; 1 | 41; 4 | 437 |
| *GCGS037; ERR191766 | 2 | 0.125 | 2 | 0.5 | **266** | **13** | **517** | 13; **3** | 32 | **14** | **104** | 16 |  | 0.5; 1 | truncated; 4 | 437 |
| GCGS038; ERR191767 | 0.5 | 0.008 | 0.015 | 0.015 | 166 | 8 | 513 | **367;** 4 | 0.015 | 230 | 242 | 1 |  | 8; 1 | 41; 3 | **431** |
| *GCGS039; ERR191768 | 4 | 0.125 | 2 | 0.25 | **266** | **13** | **550** | 13; **3** | 16 | **14** | **104** | 4 |  | 1; 1 | 41; 4 | 387 |
| GCGS040; ERR191769 | 16 | 0.03 | 0.125 | 0.03 | 291; **3** | **13** | **547** | 13; **3** | 16 | **234** | **253** | 16 | **1** | 0.125; 1 | 41; 3 | 430 |
| *GCGS041; ERR191770 | 4 | 0.125 | 2 | 0.25 | **266** | **13** | **517** | 13; **3** | 16 | **14** | **104** | 1 |  | 1; 1 | 41; 4 | 437 |
| *GCGS042; ERR191771 | 4 | 0.06 | 0.125 | 0.06 | 288 | **13** | **554** | 13; **3** | 8 | **14** | **104** | 4 |  | 1; 1 | 1531; 4 | 10 |
| *GCGS043; ERR191772 | 4 | 0.06 | 1 | 0.25 | **266** | **13** | **550** | 13; **3** | 16 | **14** | **104** | 2 |  | 1; 1 | truncated; 4 | 387 |
| GCGS044; ERR191773 | 4 | 0.06 | 0.125 | 0.06 | 283 | **13** | **526** | 13; **3** | 16 | **231** | **243** | 4 |  | 0.5; 1 | 40; 3 | 231 |
| *GCGS045; ERR191774 | 2 | 0.06 | 0.5 | 0.25 | **266** | **13** | **550** | 13; **3** | 16 | **14** | **104** | 2 |  | 1; 1 | 41; 4 | 387 |
| *GCGS046; ERR191775 | 4 | 0.03 | 0.5 | 0.06 | **266** | **13** | **550** | 13; **3** | 16 | **14** | **104** | 4 |  | 1; 1 | truncated; 4 | 387 |
| *GCGS047; ERR191776 | 4 | 0.06 | 1 | 0.25 | **266** | **13** | **550** | 13; **3** | 16 | **14** | **254** | 4 |  | 1; 1 | truncated; 4 | 387 |
| *GCGS048; ERR191777 | 4 | 0.06 | 1 | 0.03 | **266** | **13** | **550** | 13; **3** | 16 | **14** | **104** | 4 |  | 1; 1 | truncated; 4 | 387 |
| *GCGS049; ERR191778 | 2 | 0.06 | 1 | 0.25 | **266** | **13** | **550** | 13; **3** | 16 | **14** | **104** | 4 |  | 1; 1 | 1528; 4 | 387 |
| *GCGS050; ERR191779 | 0.25 | 0.008 | 0.015 | 0.015 | 20 | 8 | 543 | 192; 6 | 0.015 | 238 | 161 | 0.25 |  | 0.03; 1 | 1529; 3 | 440 |
| *GCGS051; ERR191780 | 2 | 0.06 | 1 | 0.25 | **266** | **13** | **550** | 13; **3** | 16 | **14** | **104** | 2 |  | 0.5; 1 | 41; 4 | 387 |
| *GCGS052; ERR191781 | 2 | 0.06 | 0.125 | 0.03 | 288 | **225** | **554** | 13; **3** | 8 | **14** | **104** | 2 |  | 0.5; 1 | 40; 4 | 430 |
| GCGS053; ERR191782 | 2 | 0.125 | 1 | 0.25 | **266** | **13** | **570** | 13; **3** | 16 | **14** | **104** | 2 |  | 1; 1 | 41; 4 | 437 |
| *GCGS054; ERR191783 | 2 | 0.06 | 0.125 | 0.06 | 288 | **13** | **554** | 13; **3** | 16 | **14** | **104** | 4 |  | 1; 1 | 1531; 4 | 10 |
| *GCGS055; ERR191784 | 2 | 0.06 | 1 | 0.25 | **266** | **13** | **561** | 13; **3** | 16 | **14** | **104** | 2 |  | 1; 1 | 1528; 4 | 441 |
| *GCGS056; ERR191785 | 2 | 0.06 | 0.06 | 0.06 | 21 | **13** | **579** | 13; **3** | 8 | **231** | **243** | 2 |  | 0.5; 1 | 40; 3 | 231 |
| *GCGS057; ERR191786 | 2 | 0.06 | 1 | 0.25 | **266** | **13** | **569** | 13; **3** | 16 | **14** | **104** | 2 |  | 0.5; 1 | 1528; 4 | 437 |
| *GCGS058; ERR191787 | 2 | 0.03 | 0.125 | 0.03 | 288 | **13** | **574** | 188; **3** | 16 | **14** | **104** | 2 |  | 0.5; 1 | 41; 4 | truncated |
| *GCGS059; ERR191788 | 4 | 0.125 | 2 | 0.25 | **266** | **13** | **566** | 13; **3** | 16 | **14** | **104** | 4 |  | 1; 1 | 41; 4 | 437 |
| GCGS060; ERR191789 | 4 | 0.06 | 0.125 | 0.06 | 283 | **13** | **524** | 13; **3** | 8 | truncated | **243** | 2 |  | 0.5; 1 | 40; 3 | 231 |
| *GCGS061; ERR191790 | 4 | 0.06 | 1 | 0.5 | **266** | **13** | **569** | 13; **3** | 32 | **14** | **104** | 2 |  | 0.5; 1 | 41; 4 | truncated |
| GCGS062; ERR191791 | 0.5 | 0.015 | 0.03 | 0.015 | 166 | 8 | 512 | 187; 6 | 4 | **231** | **243** | 1 |  | 0.25; 1 | 41; 3 | 430 |
| *GCGS063; ERR191792 | 2 | 0.06 | 1 | 0.25 | **266** | **13** | **542** | 13; **3** | 16 | **14** | **104** | 4 |  | 0.5; 1 | 1528; 4 | 387 |
| GCGS064; ERR191793 | 4 | 0.03 | 0.25 | 0.06 | 285 | **48** | **530** | 190; 6 | 0.015 | 232 | 244 | 32 | **2** | 1; 1 | 41; 3 | 430 |
| *GCGS065; ERR191794 | 2 | 0.06 | 1 | 0.25 | **266** | **13** | **542** | 13; **3** | 16 | **14** | **104** | 4 |  | 0.5; 1 | 1528; 4 | truncated |
| ^a^GCGS066; ERR191795 | 1 | 0.008 | 0.125 | 0.015 | **289** | **13** | **552** | 13; **3** | 8 | **14** | **104** | 2 |  | 1; 1 | 1528; 4 | 437 |
| *GCGS067; ERR191796 | 4 | 0.125 | 1 | 0.25 | **266** | **13** | **573** | 13; **3** | 16 | **14** | **104** | 0.5 |  | 0.5; 1 | 1528; 4 | 387 |
| *GCGS068; ERR191797 | 1 | 0.008 | 0.06 | 0.015 | **289** | **13** | **553** | 13; **3** | 8 | **14** | **104** | 32 |  | 0.5; 1 | 41; 4 | 437 |
| GCGS069; ERR191798 | 4 | 0.125 | 1 | 0.25 | **266** | **13** | **557** | 13; **3** | 16 | **14** | **104** | 4 |  | 1; 1 | 41; 4 | 437 |
| *GCGS070; ERR191799 | 1 | 0.015 | 0.06 | 0.03 | 288 | **13** | **587** | 13; **3** | 4 | **14** | **104** | 2 |  | 0.5; 1 | 41; 4 | 438 |
| *GCGS071; ERR191800 | 0.5 | 0.03 | 0.5 | 0.25 | **266** | 8 | 567 | 12; 6 | 0.015 | 230 | 242 | 4 |  | 0.5; 1 | 41; 3 | 430 |
| ^a^GCGS072; ERR191801 | 0.5 | 0.008 | 0.03 | 0.015 | 20 | **13** | 591 | 12; 6 | 0.015 | 240 | 259 | 2 | TetM -ve plasmid | 0.5; 1 | 40; 3 | 387 |
| *GCGS073; ERR191802 | 1 | 0.06 | 0.5 | 0.25 | **266** | 8 | 549 | 12; 6 | 0.015 | 230 | 242 | 2 |  | 0.25; 1 | 41; 3 | 430 |
| GCGS074; ERR191803 | 0.5 | 0.008 | 0.03 | 0.015 | 166 | **222** | 515 | 13; **3** | 0.015 | 19 | 244 | 0.5 |  | 0.25; 1 | 41; 3 | 10 |
| *GCGS075; ERR191804 | 2 | 0.06 | 1 | 0.25 | **266** | **13** | **517** | 13; **3** | 8 | **14** | **104** | 4 |  | 0.5; 1 | 1528; 4 | truncated |
| *GCGS076; ERR191805 | 1 | 0.008 | 0.06 | 0.015 | **289** | **13** | **553** | 13; **3** | 8 | **14** | **104** | 2 |  | 0.25; 1 | 41; 4 | 387 |
| *GCGS077; ERR191806 | 4 | 0.06 | 2 | 0.25 | **266** | **13** | **571** | 13; **3** | 16 | **14** | **104** | 1 |  | 1; 1 | 1528; 4 | 437 |
| *GCGS078; ERR191807 | 0.25 | 0.008 | 0.015 | 0.015 | 21 | **13** | **568** | 13; **3** | 4 | **231** | **243** | 4 |  | 0.06; 1 | 40; 3 | 231 |
| *GCGS079; ERR191808 | 2 | 0.06 | 2 | 0.25 | **266** | **13** | **569** | 13; **3** | 16 | **14** | **104** | 4 |  | 0.5; 1 | 1528; 4 | 387 |
| *GCGS080; ERR191809 | 4 | 0.06 | 0.125 | 0.015 | 288 | **13** | **564** | 13; **3** | 16 | **14** | **104** | 4 |  | 1; 1 | 41; 4 | 10 |
| *GCGS081; ERR191810 | 2 | 0.06 | 1 | 0.25 | **266** | **13** | **569** | 13; **3** | 16 | **14** | **104** | 2 |  | 0.125; 1 | truncated; 4 | 387 |
| GCGS082; ERR191811 | 2 | 0.06 | 0.125 | 0.06 | 287 | **13** | **539** | **370**; 6 | 16 | **237** | **243** | 2 | TetM -ve plasmid | 0.25; 1 | 40; 3 | 430 |
| GCGS083; ERR191812 | 2 | 0.03 | 0.125 | 0.03 | 166 | 8 | **517** | 13; **3** | 0.03 | 230 | 242 | 2 |  | 0.5; 1 | 41; 3 | 430 |
| GCGS084; ERR191813 | 2 | 0.015 | 0.25 | 0.015 | 166 | 8 | **517** | 13; **3** | 0.015 | 230 | 242 | 4 |  | 1; 1 | 41; 3 | 430 |
| *GCGS085; ERR191814 | 2 | 0.03 | 1 | 0.25 | **266** | **13** | **581** | 13; **3** | 32 | **14** | **104** | 4 |  | 0.5; 1 | 1535; 4 | 387 |
| *GCGS086; ERR191815 | 2 | 0.015 | 0.03 | 0.03 | truncated | **13** | 559 | 13; | 4 | **14** | **104** | 1 |  | 1; 1 | 41; 4 | 10 |
| *GCGS087; ERR191816 | 2 | 0.03 | 1 | 0.25 | **266** | **13** | **560** | 13; **3** | 32 | **14** | **104** | 4 |  | 0.5; 1 | 41; 4 | 437 |
| *GCGS088; ERR191817 | 0.5 | 0.008 | 0.015 | 0.125 | **266** | **13** | **560** | 13; **3** | 0.015 | **14** | **104** | 0.5 |  | 0.5; 1 | 1528; 4 | 437 |
| *GCGS089; ERR191818 | 2 | 0.03 | 2 | 0.25 | **266** | **13** | **517** | 13; **3** | 32 | **14** | **104** | 2 |  | 1; 1 | 41; 4 | 437 |
| GCGS090; ERR191819 | 8 | 0.008 | 0.03 | 0.015 | 228; truncated | **13** | 535 | **368**; 6 | 1 | **234** | **246** | 32 | **2** | 0.25; 1 | 40; 3 | 234 |
| *GCGS091; ERR191820 | 4 | 0.06 | 1 | 0.25 | **266** | **13** | **551** | 13; **3** | 16 | **14** | **104** | 4 |  | 1; 1 | 41; 4 | 387 |
| GCGS092; ERR191821 | 1 | 0.06 | 0.5 | 0.06 | 166 | **13** | 521 | 187; 6 | 0.015 | 19 | 242 | 1 |  | 0.25; 1 | 41; 3 | 430 |
| *GCGS093; ERR191822 | 4 | 0.125 | 1 | 0.25 | **266** | **13** | **551** | 13; **3** | 16 | **14** | **104** | 4 |  | 1; 1 | 41; 4 | 387 |
| *GCGS094; ERR191823 | 2 | 0.03 | 0.125 | 0.03 | 288 | **13** | **588** | 13; **3** | 8 | **14** | **104** | 1 |  | 1; 1 | 41; 4 | 446 |
| *GCGS095; ERR191824 | 2 | 0.06 | 1 | 0.25 | **266** | **13** | **517** | 13; **3** | 16 | **14** | **104** | 2 |  | 1; 1 | 41; 4 | truncated |
| GCGS096; ERR191825 | 0.5 | 0.008 | 0.03 | 0.03 | 166 | 8 | 513 | 187; 6 | 0.015 | 230 | 242 | 1 |  | 16; 1 | 41; 3 | **431** |
| *GCGS097; ERR223603 | 2 | 0.125 | 2 | 0.5 | **266** | **13** | **517** | 13; **3** | 16 | **14** | **104** | 4 |  | 0.5; 1 | 41; 4 | 387 |
| GCGS098; ERR223604 | 0.25 | 0.008 | 0.03 | 0.03 | 166 | 8 | 513 | 187; 6 | 0.015 | 230 | 242 | 1 |  | 16; 1 | 41; 3 | **431** |
| *GCGS099; ERR223605 | 4 | 0.25 | 2 | 0.5 | **266** | **13** | **530** | 188; **3** | 16 | **14** | **104** | 1 |  | 1; 1 | 41; 4 | 445 |
| *GCGS100; ERR223606 | 2 | 0.06 | 0.25 | 0.06 | 286 | **48** | **562** | 12; 6 | 0.015 | 232 | 244 | 1 |  | 0.25; 1 | 41; 3 | 430 |
| *GCGS101; ERR223607 | 2 | 0.06 | 2 | 0.25 | **266**; **2** | **13** | **552** | 13; **3** | 16 | **14** | **104** | 4 |  | 0.125; 1 | 41; 4 | 437 |
| *GCGS102; ERR223608 | 4 | 0.03 | 0.5 | 0.125 | **266** | **13** | **541** | 13; **3** | 16 | **14** | **104** | 2 |  | 2; 1 | 41; 4 | **439** |
| *GCGS103; ERR223609 | 2 | 0.03 | 1 | 0.25 | **266**; **2** | **13** | **517** | 13; **3** | 16 | **14** | **104** | 2 |  | 0.5; 1 | 41; 4 | 437 |
| *GCGS104; ERR223610 | 4 | 0.03 | 0.5 | 0.125 | **266** | **13** | **517** | 13; **3** | 16 | **14** | **104** | 4 |  | 2; 1 | 41; 4 | **439** |
| *GCGS105; ERR223611 | 1 | 0.06 | 1 | 0.25 | **266** | **13** | **546** | 13; **3** | 16 | **14** | **104** | 2 |  | 0.5; 1 | 41; 4 | 387 |
| ^a^GCGS106; ERR223612 | 0.5 | 0.008 | 0.03 | 0.015 | 166 | 8 | 523 | 12; 6 | 0.015 | truncated | 242 | 0.5 |  | 8; 1 | 1534; 3 | **431** |
| *GCGS107; ERR223613 | 4 | 0.125 | 0.5 | 0.25 | **266** | **13** | **517** | 13; **3** | 16 | **14** | **104** | 3 |  | 0.5; 1 | 1528; 4 | 437 |
| *GCGS108; ERR223614 | 2 | 0.06 | 0.125 | 0.03 | 288 | 226 | **577** | 188; **3** | 16 | **14** | **104** | 4 |  | 0.25; 1 | 41; 4 | 438 |
| *GCGS109; ERR223615 | 4 | 0.06 | 2 | 0.25 | **266** | **13** | **517** | 13; **3** | 32 | **14** | **104** | 4 |  | 0.5; 1 | 41; 4 | 387 |
| GCGS110; ERR223616 | 0.25 | 0.008 | 0.015 | 0.03 | 282 | 8 | 513 | 187; 6 | 0.015 | 230 | 242 | 1 |  | 16; 1 | 41; 3 | **431** |
| *GCGS113; ERR223619 | 2 | 0.03 | 2 | 0.25 | **266** | **13** | **540** | 13; **3** | 32 | **14** | **104** | 4 |  | 0.5; 1 | 1528; 4 | 437 |
| *GCGS114; ERR223620 | 0.25 | 0.008 | 0.015 | 0.015 | **289** | **13** | **545** | 13; **3** | 2 | **14** | **104** | 0.5 |  | 0.125; 1 | 1528; 4 | 437 |
| *GCGS115; ERR223621 | 4 | 0.06 | 2 | 0.5 | **266** | **13** | **517** | 13; **3** | 16 | truncated | **104** | 4 |  | 0.5; 1 | 1528; 4 | 387 |
| *GCGS116; ERR223622 | 0.25 | 0.008 | 0.015 | 0.015 | 21 | **13** | **544** | 13; **3** | 4 | **231** | **255** | 0.5 |  | 0.06; 1 | 40; 3 | 231 |
| *GCGS117; ERR223623 | 1 | 0.06 | 1 | 0.25 | **266** | **13** | **517** | 13; **3** | 16 | **14** | **104** | 2 |  | 0.25; 1 | 41; 4 | 387 |
| GCGS118; ERR223624 | 0.25 | 0.03 | 0.03 | 0.03 | 166 | 8 | 522 | 187; 6 | 0.015 | 230 | 242 | 1 |  | 8; 1 | 41; 3 | **431** |
| *GCGS119; ERR223625 | 1 | 0.06 | 1 | 0.25 | **266** | **13** | **517** | 13; **3** | 16 | **14** | **104** | 2 |  | 0.25; 1 | 41; 4 | 387 |
| GCGS120; ERR223626 | 0.25 | 0.008 | 0.015 | 0.015 | 166 | 8 | 525 | 187; 6 | 0.015 | 230 | 242 | 0.5 |  | 8; 1 | 41; 3 | **431** |
| *GCGS121; ERR223627 | 2 | 0.06 | 4 | 0.5 | **266** | **13** | **552** | 13; **3** | 16 | **14** | **104** | 2 |  | 0.5; 1 | 41; 4 | 387 |
| *GCGS122; ERR223628 | 2 | 0.03 | 0.25 | 0.06 | 288 | 226 | **577** | 188; **3** | 16 | **14** | **104** | 2 |  | 0.25; 1 | 41; 4 | 438 |
| *GCGS123; ERR223629 | 2 | 0.125 | 2 | 0.25 | truncated | **13** | **552** | 13; | 16 | **14** | **104** | 2 |  | 0.5; 1 | 41; 4 | 387 |
| *GCGS124; ERR223630 | 0.25 | 0.008 | 0.015 | 0.125 | **266** | **13** | **517** | 13; **3** | 0.015 | **14** | **104** | 0.25 |  | 0.03; 1 | 41; 4 | 437 |
| *GCGS125; ERR223631 | 4 | 0.06 | 2 | 0.25 | **266** | **13** | **552** | 13; **3** | 16 | **14** | **104** | 4 |  | 0.5; 1 | 41; 4 | 387 |
| GCGS126; ERR223632 | 0.5 | 0.03 | 0.125 | 0.125 | **281** | 8 | 516 | 187; 6 | 0.015 | truncated | 242 | 0.5 |  | 0.25; 1 | 41; 3 | 430 |
| *GCGS127; ERR223633 | 1 | 0.03 | 1 | 0.25 | **266** | **13** | 558 | 13; **3** | 8 | truncated | **104** | 0.5 |  | 0.25; 1 | 41; 4 | 437 |
| GCGS128; ERR223634 | 0.25 | 0.008 | 0.03 | 0.03 | 166 | 8 | 513 | **367**; 4 | 0.015 | 230 | 242 | 0.5 |  | 8; 1 | 41; 3 | **431** |
| *GCGS129; ERR223635 | 2 | 0.03 | 1 | 0.25 | **266** | **13** | **517** | 13; **3** | 32 | **14** | **104** | 4 |  | 0.5; 1 | 1532; 4 | 387 |
| *GCGS130; ERR223636 | 2 | 0.03 | 0.125 | 0.03 | 288 | 226 | **577** | 188; **3** | 16 | **14** | **104** | 4 |  | 0.5; 1 | 41; 4 | 438 |
| *GCGS131; ERR223637 | 2 | 0.03 | 1 | 0.25 | **266** | **13** | **517** | 13; **3** | 32 | **14** | **104** | 0.5 |  | 0.5; 1 | 1533; 4 | 437 |
| *GCGS132; ERR223638 | 4 | 0.03 | 0.125 | 0.06 | 288 | **225** | **554** | 13; **3** | 16 | **14** | **104** | 2 |  | 0.5; 1 | 40; 4 | 430 |
| *GCGS133; ERR223639 | 2 | 0.03 | 1 | 0.25 | **266** | **13** | **552** | 13; **3** | 32 | **14** | **104** | 2 |  | 0.5; 1 | 41; 4 | 387 |
| *GCGS134; ERR223640 | 4 | 0.03 | 0.125 | 0.03 | 21 | **13** | **554** | 13; **3** | 16 | **231** | **243** | 2 |  | 0.5; 1 | 40; 3 | 231 |
| *GCGS135; ERR223641 | 2 | 0.03 | 1 | 0.5 | **266** | **13** | **552** | 13; **3** | 16 | **14** | **104** | 4 |  | 0.5; 1 | 41; 4 | 437 |
| GCGS136; ERR223642 | 0.25 | 0.008 | 0.03 | 0.03 | 166 | 8 | 520 | **367**; 4 | 0.015 | 230 | 242 | 1 |  | 8; 1 | 41; 3 | **431** |
| *GCGS137; ERR223643 | 2 | 0.06 | 1 | 0.25 | **266** | **13** | **517** | 13; **3** | 32 | **14** | **104** | 2 |  | 0.5; 1 | 41; 4 | 437 |
| GCGS138; ERR223644 | 0.25 | 0.008 | 0.015 | 0.015 | 166 | 8 | 513 | **367**; 4 | 0.015 | 230 | 242 | 1 |  | 16; 1 | 41; 3 | **431** |
| *GCGS139; ERR223645 | 4 | 0.125 | 2 | 0.25 | **266** | **13** | **552** | 13; **3** | 32 | **14** | **104** | 4 |  | 1; 1 | 41; 4 | 387 |
| *GCGS140; ERR223646 | 4 | 0.06 | 0.25 | 0.06 | 288 | 226 | **577** | 188; **3** | 32 | **14** | **104** | 4 |  | 1; 1 | 41; 4 | truncated |
| *GCGS141; ERR223647 | 4 | 0.06 | 1 | 0.25 | **266** | **13** | **550** | 13; **3** | 16 | **14** | **254** | 2 |  | 0.5; 1 | 41; 4 | 387 |
| *GCGS142; ERR223648 | 0.5 | 0.008 | 0.015 | 0.015 | 285 | **13** | **531** | 13; **3** | 0.015 | 19 | 248 | 1 |  | 2; 1 | 1527; 3 | **436** |
| *GCGS143; ERR223649 | 4 | 0.125 | 1 | 0.25 | **266** | **13** | **550** | 13; **3** | 16 | **14** | **254** | 2 |  | 0.5; 1 | 41; 4 | 387 |
| GCGS144; ERR223650 | 0.5 | 0.03 | 0.25 | 0.06 | 166 | 8 | 514 | 12; 6 | 0.015 | 230 | 242 | 1 |  | 0.5; 1 | 41; 3 | 430 |
| *GCGS145; ERR223651 | 4 | 0.06 | 1 | 0.25 | **266** | **13** | **517** | 13; **3** | 16 | **14** | **104** | 1 |  | 1; 1 | 41; 4 | 437 |
| GCGS146; ERR223652 | 4 | 0.008 | 0.06 | 0.03 | 228; truncated | **13** | 537 | **368**; 6 | 1 | **234** | **246** | 16 | **2** | 0.25; 1 | 40; 3 | 234 |
| *GCGS147; ERR223653 | 2 | 0.125 | 2 | 0.25 | **266** | **13** | **550** | 13; **3** | 16 | **14** | **104** | 4 |  | 1; 1 | 41; 4 | 387 |
| *GCGS148; ERR223654 | 2 | 0.015 | 0.03 | 0.03 | 288 | **13** | 559 | 13; **3** | 4 | truncated | **104** | 1 |  | 0.5; 1 | 41; 4 | 10 |
| *GCGS149; ERR223655 | 1 | 0.03 | 0.25 | 0.25 | **266** | 8 | 549 | 12; 6 | 0.015 | 230 | 242 | 2 |  | 0.5; 1 | 41; 3 | 430 |
| *GCGS150; ERR223656 | 16 | 0.015 | 0.03 | 0.015 | 285; **3** | **13** | **594** | 13; **3** | 8 | **14** | 261 | 4 | **1** | 0.5; 1 | 40; 3 | 231 |
| *GCGS151; ERR223657 | 2 | 0.06 | 1 | 0.25 | **266** | **13** | **517** | 13; **3** | 16 | truncated | **104** | 2 |  | 1; 1 | 1528; 4 | 387 |
| *GCGS152; ERR223658 | 2 | 0.06 | 0.25 | 0.06 | 290 | **13** | **547** | 13; **3** | 16 | **14** | **252** | 2 |  | 0.5; 1 | 41; 3 | 387 |
| *GCGS153; ERR223659 | 0.5 | 0.03 | 0.25 | 0.25 | **266** | 8 | 549 | 12; 6 | 0.015 | 230 | 242 | 0.5 |  | 0.5; 1 | 41; 3 | 430 |
| GCGS154; ERR223660 | 32 | 0.008 | 0.015 | 0.015 | 23; **2** | 223 | 536 (PIA) | **369**; 6 | 4 | **233** | 249 | 16 | **1** | 0.5; 1 | 41; 3 | 234 |
| *GCGS155; ERR223661 | 4 | 0.125 | 2 | 0.25 | **266** | **13** | **517** | 13; **3** | 16 | **14** | **104** | 4 |  | 1; 1 | 41; 4 | 437 |
| GCGS156; ERR223662 | 0.25 | 0.008 | 0.03 | 0.03 | 166 | 8 | 519 | 187; 6 | 0.015 | 230 | 242 | 1 |  | 16; 1 | 41; 3 | **432** |
| ***GCGS157**; ERR223663 | 8 | 0.125 | 0.5 | 0.25 | **266** | **13** | **517** | 13; **3** | 16 | **14** | **104** | 4 |  | 1; 1 | 41; 4 | 437 |
| *GCGS158; ERR223664 | 4 | 0.03 | 0.125 | 0.03 | 288 | **13** | **530** | 188; **3** | 16 | **14** | **104** | 4 |  | 0.25; 1 | 41; 4 | truncated |
| *GCGS159; ERR223665 | 2 | 0.06 | 1 | 0.25 | **266** | **13** | **552** | 13; **3** | 8 | **14** | **104** | 2 |  | 0.5; 1 | 41; 4 | 437 |
| *GCGS160; ERR223666 | 0.25 | 0.008 | 0.06 | 0.015 | **289** | **13** | **552** | 13; **3** | 8 | **14** | **104** | 2 |  | 1; 1 | 1528; 4 | 387 |
| *GCGS161; ERR223667 | 0.5 | 0.03 | 0.5 | 0.25 | **266** | 8 | 549 | 12; 6 | 0.015 | 230 | 242 | 0.5 |  | 0.5; 1 | 41; 3 | 430 |
| ^b^**GCGS162**; ERR223668 | 1 | 0.008 | 0.015 | 0.015 | 294; truncated | 8 | 592 | **371**; 6 | 0.015 | 19 | 260 | 0.25 | TetM -ve plasmid | 0.5; 1 | 40; 3 | 231 |
| *GCGS163; ERR223669 | 4 | 0.25 | 2 | 0.25 | **266** | **13** | **556** | 13; **3** | 16 | **14** | **104** | 4 |  | 0.5; 1 | 1528; 4 | 441 |
| *GCGS164; ERR223670 | 4 | 0.03 | 0.125 | 0.03 | 21 | **224** | **548** | 13; **3** | 16 | **231** | **243** | 2 |  | 0.25; 1 | 40; 3 | 231 |
| *GCGS165; ERR223671 | 2 | 0.06 | 1 | 0.25 | truncated | **13** | **517** | 13; **3** | 16 | **14** | **104** | 1 |  | 1; 1 | 41; 4 | 437 |
| *GCGS166; ERR223672 | 1 | 0.008 | 0.06 | 0.015 | **289** | **13** | **586** | 13; **3** | 8 | **14** | **104** | 4 |  | 1; 1 | 1528; 4 | 387 |
| *GCGS167; ERR223673 | 1 | 0.03 | 0.5 | 0.25 | **266** | 8 | 549 | 12; 6 | 0.015 | 230 | 242 | 1 |  | 0.5; 1 | 41; 3 | 430 |
| *GCGS168; ERR223674 | 4 | 0.06 | 0.25 | 0.06 | 21 | **13** | **544** | 13; **3** | 16 | **231** | **251** | 4 |  | 1; 1 | 1530; 3 | 231 |
| *GCGS169; ERR223675 | 1 | 0.03 | 0.5 | 0.25 | **266** | 8 | 549 | 12; 6 | 0.015 | 230 | 242 | 1 |  | 0.5; 1 | 41; 3 | 430 |
| *GCGS170; ERR223676 | 4 | 0.06 | 0.25 | 0.06 | 21 | **13** | **544** | 13; **3** | 16 | **231** | **251** | 4 |  | 0.5; 1 | 1530; 3 | 231 |
| *GCGS171; ERR223677 | 1 | 0.06 | 0.5 | 0.25 | **266** | 8 | 549 | 12; 6 | 0.015 | 230 | 242 | 1 |  | 0.5; 1 | 41; 3 | 430 |
| GCGS172; ERR223678 | 1 | 0.008 | 0.03 | 0.015 | 285; **2** | **48** | 527 | 12; 6 | 0.015 | 232 | 244 | 1 |  | 2; 1 | 41; 3 | 433 |
| *GCGS173; ERR223679 | 1 | 0.06 | 0.5 | 0.25 | **266** | 8 | 549 | 12; 6 | 0.015 | 230 | 242 | 1 |  | 1; 1 | 41; 3 | 430 |
| GCGS174; ERR223680 | 0.25 | 0.008 | 0.015 | 0.03 | 166 | 8 | 513 | 187; 6 | 0.015 | 230 | 242 | 1 |  | 16; 1 | 41; 3 | **431** |
| *GCGS175; ERR223681 | 1 | 0.06 | 0.5 | 0.25 | **266** | 8 | 549 | 12; 6 | 0.015 | 230 | 242 | 1 |  | 0.5; 1 | 41; 3 | 430 |
| GCGS176; ERR223682 | 0.25 | 0.008 | 0.015 | 0.015 | 166 | 8 | 518 | 187; 6 | 0.015 | 230 | 242 | 1 |  | 16; 1 | 41; 3 | **431** |
| *GCGS177; ERR223683 | 1 | 0.03 | 0.25 | 0.25 | **266** | **13** | 582 (PIA) | 13; **3** | 4 | truncated | **104** | 4 |  | 1; 1 | 41; 4 | 437 |
| *GCGS178; ERR223684 | 1 | 0.015 | 0.25 | 0.03 | 289 | **13** | **580** | 13; **3** | 16 | **14** | **104** | 4 |  | 1; 1 | 1528; 4 | 387 |
| *GCGS179; ERR223685 | 2 | 0.06 | 1 | 0.5 | **266** | **13** | **542** | 13; **3** | 16 | **14** | **104** | 2 |  | 0.25; 1 | 1528; 4 | 387 |
| GCGS180; ERR223686 | 1 | 0.015 | 0.06 | 0.015 | 228 | **13** | **528** | **368**; 6 | 2 | **234** | **246** | 16 | **2** | 0.25; 1 | 40; 3 | 434 |
| *GCGS181; ERR223687 | 1 | 0.125 | 1 | 0.5 | **266** | **13** | **542** | 13; **3** | 16 | **14** | **104** | 2 |  | 0.25; 1 | 1528; 4 | 387 |
| *GCGS182; ERR223688 | 1 | 0.008 | 0.03 | 0.015 | 23 | **13** | 572 | 13; **3** | 4 | **14** | **104** | 2 |  | 0.5; 1 | 41; 4 | 10 |
| *GCGS183; ERR223689 | 1 | 0.06 | 1 | 0.25 | **266**; **2** | **13** | **542** | 13; **3** | 16 | **14** | **104** | 2 |  | 0.25; 1 | 1528; 4 | 387 |
| GCGS184; ERR223690 | 16 | 0.008 | 0.015 | 0.015 | 23; truncated | 227 | 563 | 193; 6 | 2 | **233** | 249 | 0.25 | TetM -ve plasmid | 0.03; 1 | 40; 3 | 442 |
| *GCGS185; ERR223691 | 2 | 0.06 | 1 | 0.25 | **266** | **13** | **542** | 13; **3** | 16 | **14** | **104** | 4 |  | 0.25; 1 | 41; 4 | 437 |
| GCGS186; ERR223692 | 8 | 0.03 | 0.06 | 0.03 | 228; truncated | **13** | **534** | **368**; 6 | 2 | **234** | **246** | 64 | **2** | 0.25; 1 | 40; 3 | 234 |
| *GCGS187; ERR223693 | 2 | 0.125 | 1 | 0.25 | **266** | **13** | **542** | 13; **3** | 16 | **14** | **104** | 2 |  | 0.25; 1 | 1528; 4 | 387 |
| GCGS188; ERR223694 | 0.25 | 0.015 | 0.03 | 0.03 | 228 | **13** | 529 | **368**; 6 | 1 | **234** | **246** | 16 | **2** | 0.25; 1 | 40; 3 | 231 |
| *GCGS189; ERR191795 | 4 | 0.06 | 2 | 0.25 | **266** | **13** | **575** | 13; **3** | 16 | **14** | **104** | 4 |  | 1; 1 | 41; 4 | 387 |
| *GCGS190; ERR223696 | 4 | 0.03 | 0.25 | 0.03 | 21 | **13** | **544** | 13; **3** | 16 | **231** | **243** | 0.25 |  | 1; 1 | 1530; 3 | 231 |
| *GCGS191; ERR223697 | 1 | 0.03 | 0.5 | 0.25 | **266** | 8 | 549 | 12; 6 | 0.015 | 230 | 242 | 1 |  | 0.5; 1 | 41; 3 | 430 |
| GCGS192; ERR223698 | 0.5 | 0.008 | 0.03 | 0.03 | 166 | 8 | 513 | 13; **3** | 0.015 | 230 | 242 | 1 |  | 2; 1 | 41; 3 | 430 |
| *GCGS193; ERR222892 | 0.5 | 0.03 | 0.5 | 0.25 | **266** | 8 | 549 | 12; 6 | 0.015 | 230 | 242 | 1 |  | 0.5; 1 | 41; 3 | 430 |
| *GCGS194; ERR222893 | 1 | 0.03 | 0.06 | 0.015 | 288 | **13** | **587** | 13; **3** | 4 | **14** | **104** | 1 |  | 0.5; 1 | 41; 4 | 438 |
| *GCGS195; ERR222894 | 4 | 0.06 | 1 | 0.25 | **266** | **13** | **552** | 13; **3** | 16 | **14** | **104** | 4 |  | 0.5; 1 | 41; 4 | 387 |
| *GCGS196; ERR222895 | 4 | 0.03 | 0.25 | 0.03 | 288 | **13** | **635** | 13; **3** | 16 | truncated | **104** | 4 |  | 1; 1 | 41; 4 | 10 |
| *GCGS197; ERR222896 | 1 | 0.015 | 0.5 | 0.25 | **266** | 8 | 549 | 12; 6 | 0.015 | 230 | 242 | 1 |  | 0.5; 1 | 41; 3 | 430 |
| GCGS198; ERR222897 | 0.25 | 0.008 | 0.015 | 0.015 | 166 | 8 | 525 | 187; 6 | 0.015 | 230 | 242 | 1 |  | 16; 1 | 41; 3 | **431** |
| *GCGS199; ERR222898 | 1 | 0.015 | 0.5 | 0.25 | **266** | 8 | 549 | 12; 6 | 0.015 | 230 | 242 | 1 |  | 0.5; 1 | 41; 3 | 430 |
| ^b^GCGS200; ERR222899 | 0.25 | 0.015 | 0.015 | 0.015 | 17 | 8 | 630 | 189; 6 | 0.015 | 235 | 247 | 0.25 |  | 0.125; 1 | 40; 3 | 435 |
| *GCGS201; ERR222900 | 1 | 0.015 | 0.5 | 0.25 | **266** | 8 | 549 | 12; 6 | 0.015 | 230 | 242 | 2 |  | 0.5; 1 | 41; 3 | 430 |
| GCGS202; ERR222901 | 0.25 | 0.008 | 0.015 | 0.03 | 166 | 8 | 525 | 187; 6 | 0.015 | 230 | 242 | 0.5 |  | 8; 1 | 41; 3 | **431** |
| *GCGS203; ERR222902 | 1 | 0.06 | 0.5 | 0.25 | **266** | 8 | 549 | 12; 6 | 0.015 | 230 | 242 | 2 |  | 0.5; 1 | 41; 3 | 430 |
| GCGS204; ERR222903 | 0.25 | 0.008 | 0.015 | 0.015 | 166 | 8 | 626 | 187; 6 | 0.015 | 230 | 242 | 1 |  | 8; 1 | truncated; 3 | **431** |
| *GCGS205; ERR222904 | 4 | 0.125 | 2 | 0.25 | **266** | **13** | **517** | 13; **3** | 16 | **14** | **104** | 2 |  | 1; 1 | 41; 4 | 437 |
| *GCGS206; ERR222905 | 0.25 | 0.008 | 0.03 | 0.015 | 21 | **13** | **544** | 13; **3** | 4 | truncated | **255** | 1 |  | 0.06; 1 | 40; 3 | 231 |
| *GCGS207; ERR222906 | 1 | 0.06 | 0.5 | 0.25 | **266** | 8 | 549 | 12; 6 | 0.015 | 230 | 242 | 1 |  | 0.5; 1 | 41; 3 | 430 |
| GCGS208; ERR222907 | 1 | 0.015 | 0.03 | 0.015 | 23 | 223 | **629** | 12; **3** | 8 | **233** | 245 | 2 | TetM -ve plasmid | 1; 1 | 40; 3 | 231 |
| *GCGS209; ERR222908 | 0.5 | 0.03 | 0.5 | 0.25 | **266** | 8 | 549 | 12; 6 | 0.015 | 230 | 242 | 0.5 |  | 0.5; 1 | 41; 3 | 430 |
| GCGS210; ERR222909 | 0.5 | 0.015 | 0.06 | 0.03 | 228 | **13** | 537 | **368**; 6 | 1 | **234** | **246** | 16 | **2** | 0.25; 1 | 40; 3 | 234 |
| *GCGS211; ERR222910 | 0.5 | 0.03 | 0.5 | 0.25 | **266** | 8 | 549 | 12; 6 | 0.015 | 230 | 242 | 0.5 |  | 0.25; 1 | 41; 3 | 430 |
| GCGS212; ERR222911 | 1 | 0.015 | 0.06 | 0.03 | 228 | **13** | 631 | **368**; 6 | 1 | **234** | **246** | 32 | **2** | 0.25; 1 | 40; 3 | 231 |
| *GCGS213; ERR222912 | 1 | 0.03 | 0.5 | 0.25 | **266** | 8 | 549 | 12; 6 | 0.015 | 230 | 242 | 0.5 |  | 0.5; 1 | 41; 3 | 430 |
| *GCGS214; ERR222913 | 0.5 | 0.008 | 0.03 | 0.015 | 23 | **13** | 572 | 13; **3** | 2 | **14** | **104** | 0.5 |  | 0.5; 1 | 41; 4 | 10 |
| *GCGS215; ERR222914 | 0.5 | 0.03 | 0.5 | 0.25 | **266** | 8 | 549 | 12; 6 | 0.015 | 230 | 242 | 0.5 |  | 0.25; 1 | 41; 3 | 430 |
| GCGS216; ERR222915 | 4 | 0.015 | 0.03 | 0.015 | 228; **3** | **13** | 537 | **368**; 6 | 2 | **234** | **243** | 16 | **2** | 0.25; 1 | 40; 3 | 234 |
| *GCGS217; ERR222916 | 1 | 0.03 | 0.5 | 0.25 | **266** | 8 | 549 | 12; 6 | 0.015 | 230 | 242 | 1 |  | 0.5; 1 | 41; 3 | 430 |
| GCGS218; ERR222917 | 0.25 | 0.008 | 0.015 | 0.015 | 166 | 8 | 513 | 187; 6 | 0.015 | 230 | 242 |  |  | 16; 1 | 41; 3 | **431** |
| *GCGS219; ERR222918 | 1 | 0.03 | 0.5 | 0.25 | **266** | 8 | 549 | 12; 6 | 0.015 | 230 | 242 | 1 |  | 0.5; 1 | 41; 3 | 430 |
| *GCGS220; ERR222919 | 0.25 | 0.008 | 0.03 | 0.015 | 23 | **13** | 572 | 13; **3** | 2 | **14** | **104** | 1 |  | 0.06; 1 | 41; 4 | 10 |
| *GCGS221; ERR222920 | 1 | 0.03 | 0.5 | 0.25 | **266** | 8 | 549 | 12; 6 | 0.015 | 230 | 242 | 0.5 |  | 0.5; 1 | 41; 3 | 430 |
| *GCGS222; ERR222921 | 2 | 0.03 | 0.06 | 0.015 | 288 | **13** | **632** | 13; **3** | 4 | **14** | **104** | 1 |  | 0.5; 1 | 41; 4 | 438 |
| *GCGS223; ERR222922 | 0.5 | 0.03 | 0.5 | 0.25 | **266** | 8 | 549 | 12; 6 | 0.015 | 230 | 242 | 1 |  | 0.5; 1 | 41; 3 | 430 |
| GCGS224; ERR222923 | 0.25 | 0.008 | 0.015 | 0.015 | 166 | 8 | 525 | 187; 6 | 0.015 | 230 | 242 | 1 |  | 8; 1 | 41; 3 | **431** |
| *GCGS225; ERR222924 | 1 | 0.03 | 0.5 | 0.25 | **266** | 8 | 549 | 12; 6 | 0.015 | 230 | 242 | 4 |  | 0.5; 1 | 41; 3 | 430 |
| GCGS226; ERR222925 | 0.25 | 0.008 | 0.015 | 0.015 | 166 | 8 | 625 | 187; 6 | 0.015 | 230 | 242 | 0.5 |  | 16; 1 | 41; 3 | **431** |
| *GCGS227; ERR222926 | 4 | 0.06 | 1 | 0.25 | **266** | **13** | **637** | 13; **3** | 16 | **14** | **104** | 4 |  | 1; 1 | 41; 4 | 437 |
| GCGS228; ERR222927 | 2 | 0.015 | 0.06 | 0.015 | 166 | 8 | **628** | 188; **3** | 0.015 | 230 | 242 | 4 |  | 2; 1 | 41; 3 | 430 |
| *GCGS229; ERR222928 | 2 | 0.06 | 1 | 0.25 | **266** | **13** | **637** | 13; **3** | 16 | **14** | **104** | 1 |  | 0.5; 1 | 41; 4 | 437 |
| GCGS230; ERR222929 | 0.25 | 0.008 | 0.015 | 0.015 | 166 | 8 | 518 | 187; 6 | 0.015 | 230 | 242 | 0.5 |  | 8; 1 | 41; 3 | **431** |
| *GCGS231; ERR222930 | 2 | 0.03 | 0.25 | 0.25 | **266** | **13** | **636** | 13; **3** | 32 | **14** | **104** | 1 |  | 0.25; 1 | 41; 4 | 387 |
| *GCGS232; ERR222931 | 2 | 0.03 | 0.25 | 0.06 | 288 | 226 | **577** | 188; **3** | 32 | **14** | **104** | 0.5 |  | 0.5; 1 | 41; 4 | 438 |
| *GCGS233; ERR222932 | 2 | 0.06 | 1 | 0.25 | **266** | **13** | **552** | 13; **3** | 32 | **14** | **104** | 2 |  | 0.5; 1 | 41; 4 | 437 |
| *GCGS234; ERR222933 | 2 | 0.03 | 0.25 | 0.06 | 288 | 226 | **577** | 188; **3** | 32 | **14** | **104** | 2 |  | 0.5; 1 | 41; 4 | 438 |
| *GCGS235; ERR222934 | 2 | 0.06 | 1 | 0.25 | **266** | **13** | **638** | 13; **3** | 16 | **14** | **104** | 2 |  | 0.25; 1 | 1528; 4 | 387 |
| *GCGS236; ERR222935 | 2 | 0.03 | 0.25 | 0.06 | 288 | 226 | **577** | 188; **3** | 32 | **14** | **104** | 0.5 |  | 0.5; 1 | 41; 4 | 444 |
| *GCGS237; ERR222936 | 2 | 0.06 | 1 | 0.25 | **266** | **13** | **633** | 13; **3** | 16 | **14** | **104** | 4 |  | 0.25; 1 | 41; 4 | 387 |
| *GCGS238; ERR222937 | 2 | 0.03 | 0.25 | 0.06 | 288 | **13** | **634** | 188; **3** | 32 | **14** | **104** | 4 |  | 0.5; 1 | truncated; 4 | truncated |
| *GCGS239; ERR222938 | 2 | 0.125 | 2 | 0.5 | **266** | **13** | **517** | 13; **3** | 16 | **14** | **104** | 4 |  | 0.5; 1 | 1528; 4 | 387 |
| *GCGS240; ERR222939 | 2 | 0.06 | 0.25 | 0.06 | 21 | **13** | 639 | 13; **3** | 32 | **231** | **243** | 4 |  | 0.5; 1 | 1536; 3 | 231 |
| *GCGS241; ERR222940 | 4 | 0.125 | 2 | 0.5 | **266** | **13** | **517** | 13; **3** | 16 | **14** | **104** | 2 |  | 0.5; 1 | 41; 4 | 437 |
| ***GCGS242**; ERR222941 | 0.5 | 0.015 | 0.25 | 0.125 | **266** | **13** | 558 | 13; **3** | 4 | **14** | **104** | 0.5 |  | 0.125; 1 | 41; 4 | 437 |
| ^b^CH811; SRR955973 | n/a | n/a | n/a | 0.008 | 228 | 8 | 784 (PIA) | 12; 6 | n/a | 19 | 244 | 2 |  | 0.25 | 41; 3 | 387 |
| GC1-182; SRR959017 | n/a | n/a | n/a | 0.008 | 20; **3** | 404 | 968 | 380; 6 | n/a | 232 | 244 | 4 | TetM -ve plasmid | 0.5 | 41; 3 | 430 |
| *ALB0102; SRR969395 | n/a | n/a | n/a | 0.06 | 285 | **13** | **990** | 13; **3** | n/a | truncated | **104** | 2 |  | 0.25 | 41; 4 | 444 |
| ALB0303; SRR969355 | n/a | n/a | n/a | 0.015 | 228; truncated | **13** | 979 | **368**; 6 | n/a | **234** | **246** | 16 | **2** | 0.03 | 40; 3 | 231 |
| *ALB0403; SRR969356 | n/a | n/a | n/a | 0.125 | **266** | **13** | **517** | 13; **3** | n/a | **14** | **104** | 4 |  | 1 | 41; 4 | 437 |
| ATL0103; SRR969360 | n/a | n/a | n/a | 0.015 | 20 | **13** | **971** | 12; **3** | n/a | truncated | 161 | 0.25 |  | 0.5 | 40; 3 | 437 |
| ATL0105; SRR969361 | n/a | n/a | n/a | 0.015 | 228; **3** | **13** | 537 | **368**; 6 | n/a | **234** | **246** | 0.25 | **2** | 0.06 | 40; 3 | 234 |
| ^b^ATL0108; SRR969362 | n/a | n/a | n/a | 0.015 | 294 | 8 | 980 | **371**; 6 | n/a | 19 | 260 | 0.25 | TetM -ve plasmid | 0.03 | 40; 3 | 231 |
| *ATL0117; SRR969363 | n/a | n/a | n/a | 0.015 | 20 | 362 | 981 | 12; 6 | n/a | 17 | 162 | 16 | **1** | 0.125 | 1537; 3 | 452 |
| ATL0121; SRR969364 | n/a | n/a | n/a | 0.03 | 21; truncated | **13** | **647** | 13; **3** | n/a | truncated | 398 | 1 | TetM -ve plasmid | 0.5 | **1538**; 3 | 453 |
| ***ATL0125**; SRR969365 | n/a | n/a | n/a | 0.015 | **498** | **13** | **671** | 13; **3** | n/a | truncated | **104** | 1 |  | 0.25 | 41; 4 | 387 |
| *ATL0508; SRR969366 | n/a | n/a | n/a | 0.015 | 20 | 8 | 867 | 192; 6 | n/a | truncated | 161 | 16 |  | 0.06 | 40; 3 | 454 |
| ATL0513; SRR969367 | n/a | n/a | n/a | 0.03 | 285; **9** | **48** | **982** | 12; 6 | n/a | truncated | 244 | 2 |  | 0.25 | 41; 3 | 430 |
| *MIA0202; SRR969368 | n/a | n/a | n/a | 0.03 | 21 | **13** | **983** | 13; **3** | n/a | **231** | **243** | 2 |  | 0.5 | 40; 3 | 231 |
| ^b^MIA0309; SRR969369 | n/a | n/a | n/a | 0.015 | 546; **3** | 405 | 984 (PIA) | 18; 6 | n/a | 420 | 260 | 16 | **1** | 0.125 | 40; 3 | 10 |
| ^b^MIA0310; SRR969370 | n/a | n/a | n/a | 0.015 | 294 | 8 | 727 | **371;** 6 | n/a | truncated | 260 | 16 | TetM -ve plasmid | 0.03 | 40; 3 | 231 |
| *MIA0510; SRR969373 | n/a | n/a | n/a | 0.03 | **289** | **13** | **553** | 13; 6 | n/a | **14** | **104** | 2 |  | 1 | 41; 4 | 387 |
| *MIA0515; SRR969371 | n/a | n/a | n/a | 0.03 | 288 | **13** | **530** | 188; **3** | n/a | **14** | **104** | 16 |  | 0.25 | 41; 4 | 438 |
| *MUNG12; SRR969384 | n/a | n/a | n/a | 0.25 | **266** | **13** | **517** | 13; **3** | n/a | **14** | **104** | 4 |  | 0.5 | 41; 4 | 437 |
| *MUNG14; SRR969385 | n/a | n/a | n/a | 0.25 | **547** | **13** | **552** | 13; **3** | n/a | **14** | **104** | 4 |  | 0.5 | 41; 4 | 437 |
| *MUNG15; SRR969386 | n/a | n/a | n/a | 1 | **498** | **13** | **671** | 13; **3** | n/a | **14** | **104** | 2 |  | 0.25 | 41; 4 | 387 |
| ***MUNG17**; SRR969387 | n/a | n/a | n/a | 0.5 | 22 | 8 | 986 | 329; 6 | n/a | 17 | 401 | 2 |  | 1 | 84; 3 | 234 |
| MUNG18; SRR969388 | n/a | n/a | n/a | 0.016 | 228 | **13** | 987 | 12; 6 | n/a | 386 | 431 | 2 | TetM -ve plasmid | 0.125 | 40; **83** | 437 |
| MUNG19; SRR969389 | n/a | n/a | n/a | 0.016 | 166 | 8 | 988 | 12; 6 | n/a | 230 | 242 | 2 |  | >256 | 41; 3 | **456** |
| ^b^MUNG21; SRR969391 | n/a | n/a | n/a | 0.032 | 21 | **13** | **882** | 191; 6 | n/a | **409** | 513 | 2 | TetM -ve plasmid | 1 | 1541; 3 | 231 |
| *MUNG23; SRR969392 | n/a | n/a | n/a | 0.016 | 20 | 8 | 867 | 192; 6 | n/a | 238 | 161 | 0.125 |  | 0.064 | 40; 3 | 454 |
| *MUNG25; SRR969393 | n/a | n/a | n/a | 0.016 | 288 | **13** | 989 | 13; **3** | n/a | truncated | **104** | 0.5 |  | 0.125 | 41; 4 | 437 |
| ^b^MUNG26; SRR969394 | n/a | n/a | n/a | 0.016 | 294 | 8 | 727 | **371;** 6 | n/a | 19 | 260 | 0.5 | TetM -ve plasmid | 0.064 | 40; 3 | 231 |
| MUNG3; SRR969378 | n/a | n/a | n/a | 0.5 | **14** | **13** | **810** | 12; **3** | n/a | **193** | **206** | 2 |  | 0.25 | 1539; 3 | 10 |
| **MUNG4**; SRR969379 | n/a | n/a | n/a | 0.25 | 517 | **13** | **786** | 12; 6 | n/a | **193** | 488 | 4 | TetM -ve plasmid | 0.5 | 1540; 3 | 156 |
| *MUNG5; SRR969380 | n/a | n/a | n/a | 0.016 | **511** | **13** | **729** | 13; **3** | n/a | **14** | **104** | 1 |  | 0.25 | 41; 4 | 387 |
| ^a^MUNG6; SRR969381 | n/a | n/a | n/a | 0.016 | 22 | 185 | 640 (PIA) | 194; 6 | n/a | 19 | 455 | 16 |  | 0.125 | 41; 3 | 455 |
| MUNG8; SRR969382 | n/a | n/a | n/a | 0.016 | 166 | 8 | 728 | **373;** 5 | n/a | 230 | 242 | 0.5 |  | 2 | 41; 3 | 430 |
| *MUNG9; SRR969383 | n/a | n/a | n/a | 1 | **500** | **13** | **517** | 13; **3** | n/a | truncated | **104** | 2 |  | 0.5 | 41; 4 | 437 |
| NOR0306; SRR969374 | n/a | n/a | n/a | 0.015 | 166; **3** | **13** | 699 (PIA) | **376;** 6 | n/a | **233** | **508** | 2 | **1** | 0.25 | 40; 3 | 387 |
| *NYC0507; SRR969375 | n/a | n/a | n/a | 0.06 | 288 | **13** | **530** | 188; **3** | n/a | **14** | **104** | 2 |  | 0.25 | 41; 4 | 438 |
| *NYC0513; SRR969376 | n/a | n/a | n/a | 0.06 | 21 | **13** | **985** | 13; **3** | n/a | **231** | **243** | 4 |  | 0.25 | 40; 3 | 231 |
| SK14515; SRR969340 | n/a | n/a | n/a | 0.016 | 20 | **48** | **971** | 12; 6 | n/a | truncated | 244 | 2 |  | 0.25 | 41; 3 | 430 |
| *SK15454; SRR969341 | n/a | n/a | n/a | 0.004 | 20 | 8 | 972 | 192; 6 | n/a | truncated | 161 | 2 |  | 0.06 | 40; 3 | 231 |
| SK16259; SRR969342 | n/a | n/a | n/a | 0.008 | 285 | **48** | 973 | 12; 6 | n/a | truncated | 244 | 4 |  | 0.125 | 41; 3 | 430 |
| SK16942; SRR969343 | n/a | n/a | n/a | 0.016 | 285 | **48** | **974** | 12; 6 | n/a | truncated | 244 | 2 |  | 0.125 | 41; 3 | 430 |
| SK17973; SRR969344 | n/a | n/a | n/a | 0.016 | 285 | **48** | **975** | 12; 6 | n/a | truncated | 244 | 8 |  | 1 | 41; 3 | 430 |
| ^a^SK22871; SRR969345 | n/a | n/a | n/a | 0.004 | 20 | 8 | 877 | 194; 1 | n/a | 393 | 465 | 4 | TetM -ve plasmid | 0.125 | 40; 3 | 450 |
| SK28355; SRR969348 | n/a | n/a | n/a | 0.016 | 285 | **48** | **971** | 12; 6 | n/a | truncated | 244 | 4 |  | 0.25 | 41; 3 | 430 |
| SK29471; SRR969350 | n/a | n/a | n/a | 0.016 | 285 | **48** | **975** | 12; 6 | n/a | truncated | 541 | 2 |  | 0.25 | 41; 3 | 430 |
| *SK32402; SRR969352 | n/a | n/a | n/a | 0.016 | 288 | **13** | 976 | 13; **3** | n/a | truncated | **104** | 4 |  | 0.5 | 41; 4 | 451 |
| ^b^SK33414; SRR969353 | n/a | n/a | n/a | 0.008 | 23 | **13** | 977 | 381; 6 | n/a | truncated | 106 | 4 | TetM -ve plasmid | 0.25 | 41; 3 | 11 |
| ^a^SK36809; SRR969351 | n/a | n/a | n/a | 0.008 | 166 | 8 | 523 | 12; 6 | n/a | truncated | 242 | 8 |  | 2 | 41; 3 | **431** |
| *SK39420; SRR969354 | n/a | n/a | n/a | 0.016 | 20 | 8 | 978 (PIA) | 192; 6 | n/a | truncated | 161 | 0.5 |  | 0.5 | 40; 3 | 231 |
| SK6987; SRR969045 | n/a | n/a | n/a | 0.016 | 20 | **13** | **969** | 12; **3** | n/a | truncated | 161 | 4 |  | 1 | 40; 3 | 449 |
| *SK7461; SRR969336 | n/a | n/a | n/a | 0.032 | 21 | **13** | **544** | 13; **3** | n/a | truncated | **243** | 8 |  | 0.5 | 40; 3 | 231 |
| SK7842; SRR969337 | n/a | n/a | n/a | 0.016 | 20 | **13** | **970** | 12; **3** | n/a | truncated | 161 | 8 |  | 1 | 40; 3 | 437 |

PEN: penicillin; CRO: ceftriaxone; CPD: cefpodoxime; CFM: cefixime; CIP: ciprofloxacin; AZM: azithromycin; STR: spectinomycin.

Bold underlined numbers denote alleles containing amino acid substitutions associated with conferring antibiotic resistance; n/a: antibiotic profiles to azithromycin, cefixime and tetracycline only were available for these isolates; truncated: incomplete WGS sequence;*: isolates with GGI, ^a^: isolates with *traG3*-like alleles (*N. meningitidis* GGI), ^b^: isolates with *traG2*-like alleles.

Mutations associated with resistant AMR MIC values were not identified in the efflux pumps MacAB and FarAB ([5](#_ENREF_5), [6](#_ENREF_6)). None of the isolates were found with nucleotide substitution G→T in the -10 promoter region (5’-TA**G**AAT-3’) upstream of *macA* (^pro^NEIS0488) and no significant mutations were found in the transcriptional regulator, NEIS0374 (*farR)* ([7](#_ENREF_7)). Overexpression of NEIS0763 (*norM*) may occur when a T→C nucleotide occurs in the -35 box in the promoter region (^pro^NEIS0763) (TTGACG to **C**TGACG) ([8](#_ENREF_8)) and all isolates contained this substitution.

**Supplementary Table 5. PorB loop III variable regions**

| **variable sites in loop III (bold denotes residues 120 and 121 associated with AMR)** | **VR type** | **distribution in isolates** | **MIC values where available** |
| --- | --- | --- | --- |
| LNSPLKNTGA NVNAWES--- -GKYTGEFLE ISKMARREHR | **III.1** | 41/289 (14%) | PEN: 0.25-2;  TET: 0.25-16*;  CFM: 0.015-0.25;  CRO: 0.008-0.06;  CPD: 0.015-0.5 |
| ...... .G**S** K.N....--- -G.Y..EF.E ..K..E.... | **III.2** | 5/289 (2%) | PEN: 0.5-1;  TET: 0.5-1;  CFM: 0.015-0.25;  CRO: 0.03-0.06;  CPD: 0.003-1 |
| ........GA N.N....--- -G.Y..EL.E ..K..G.... | **III.3** | 23/289 (8%) | PEN: 0.25;  TET: 0.5-1;  CFM: 0.015-0.6;  CRO: 0.008-0.03;  CPD: 0.015-0.125 |
| ........GA N.N....--- -G.Y..EF.E ..K..Q.... | **III.4** | 2/289 (0.7%) | PEN: 0.5-16*;  TET: 0.25-0.5;  CFM: 0.015;  CRO: 0.008;  CPD: 0.015-0.03 |
| ........**KN** N.N....--- -G.F..NV.E ..G..Q.... | **III.5** | 102/289 (35%) | PEN: 0.25-8;  TET: 0.25-16;  CFM: 0.015-0.5;  CRO: 0.008-0.25;  CPD: 0.015-2 |
| ........**KD** N.N....--- -G.F..NV.E ..G..K.... | **III.6** | 13/289 (5%) | PEN: 2-4;  TET: 1-4;  CFM: 0.015-0.06;  CRO: 0.015-0.06;  CPD: 0.125-0.06 |
| ........G**S** K.N....--- -G.F..NV.E ..G..E.... | **III.7** | 3/289 (1%) | PEN: 0.5-1;  TET: 1-8;  CFM: 0.015-0.25;  CRO: 0.008-0.25;  CPD: 0.03-0.25 |
| ........**KD** N.N....--- -G.F..DV.E ..G..K.... | **III.8** | 35/289 (12%) | PEN: 0.25-8;  TET: 0.25-32*;  CFM: 0.015-0.5;  CRO: 0.008-0.25;  CPD: 0.015-2 |
| ........**KG** N.N....--- -G.F..NV.E ..G..K.... | **III.9** | 8/289 (3%) | PEN: 1-16*;  TET: 0.5-32*;  CFM: 0.015-0.25;  CRO: 0.015-0.06;  CPD: 0.006-0.25 |
| ........GA N.N....--- -G.Y..EF.E ..K..E.... | **III.10** | 1/289 (0.3%) | PEN: 0.25;  TET: 0.25;  CFM: 0.015;  CRO: 0.015;  CPD: 0.015 |
| ........G**S** K.N....--- -G.Y..EL.E ..K..E.... | **III.11** | 2/289 (0.7%) | PEN: 1;  TET: 4-32*;  CFM: 0.016-0.03;  CRO: 0.015;  CPD: 0.06 |
| ........GA N.N....--- -G.Y..EF.E ..K..G.... | **III.12** | 5/289 (2%) | PEN: 0.5-4;  TET: 0.25-16;  CFM: 0.03-0.125;  CRO: 0.08-0.015;  CPD: 0.03-0.06 |
| ........GA N.N....--- -G.F..NV.E ..G..K.... | **III.13** | 2/289 (0.7%) | PEN: 0.5-2;  TET: 4-16;  CFM: 0.015-0.06;  CRO: 0.008-0.06;  CPD: 0.03-0.25 |
| ........GA N.N....--- -G.Y..KF.E ..K..G.... | **III.14** | 1/289 (0.3%) | PEN: 8*;  TET: 32*;  CFM: 0.015;  CRO: 0.008;  CPD: 0.03 |
| ........**KD** N.N....--- -G.F..NV.E ..G..Q.... | **III.15** | 3/289 (1%) | PEN: 2;  TET: 2-8;  CFM: 0.016-0.06;  CRO: 0.006;  CPD: 0.125 |
| ........**ND** N.N....--- -G.F..NV.E ..G..K.... | **III.16** | 4/289 (1.4%) | PEN: 1-2;  TET: 0.5-1;  CFM: 0.015-0.06;  CRO: 0.015-0.06;  CPD: 0.06-0.25 |
| ........**KN** N.N....--- -S.F..NV.E ..G..Q.... | **III.17** | 1/289 (0.3%) | PEN: 4;  TET: 2;  CFM: 0.06;  CRO: 0.03;  CPD: 0.5 |
| ........GA N.N....--- -G.F..NV.E ..G..Q.... | **III.18** | 5/289 (2%) | PEN: 0.25-8;  TET: 0.25-2;  CFM: 0.015-0.5;  CRO: 0.008;  CPD: 0.015 |
| ........**KD** N.N....--- -G.F..DV.E ..G..E.... | **III.19** | 1/289 (0.3%) | PEN: 2;  TET: 4;  CFM: 0.06;  CRO: 0.03;  CPD: 0.25 |
| ........**KN** N.N....--- -G.F..NV.E ..G..K.... | **III.20** | 3/289 (1%) | PEN: 2-4;  TET: 4;  CFM: 0.25;  CRO: 0.125;  CPD: 1-2 |
| ........**KN** N.N....GKF TG.F..NV.E ..G..Q.... | **III.21** | 1/289 (0.3%) | PEN: 2;  TET: 2;  CFM: 0.25;  CRO: 0.06;  CPD: 1 |
| ........G**S** K.N....--- -G.F..NV.E ..G..Q.... | **III.22** | 1/289 (0.3%) | PEN: 2;  TET: 0.5;  CFM: 0.06;  CRO: 0.06;  CPD: 1 |
| ........**RD** N.N....--- -G.F..DV.E ..G..K.... | **III.23** | 1/289 (0.3%) | PEN: 2;  TET: 1-4;  CFM: 0.008-0.032;  CRO: 0.03;  CPD: 0.06 |
| ........**D**A N.N....--- -G.F..NV.E ..G..Q.... | **III.24** | 3/289 (1%) | PEN: 2;  TET: 1-4;  CFM: 0.008-0.032;  CRO: 0.03;  CPD: 0.06 |
| ........G**S** K.N....--- -G.Y..EV.E ..G..E.... | **III.25** | 1/289 (0.3%) | PEN: n/a;  TET: 4;  CFM: 0.008;  CRO: n/a;  CPD: n/a |
| ........**NN** N.N....--- -G.F..NV.E ..G..K.... | **III.26** | 1/289 (0.3%) | PEN: n/a;  TET: 0.5  CFM: 0.016;  CRO: n/a;  CPD: n/a |
| ........**DN** N.N....--- -G.F..NV.E ..G..K.... | **III.27** | 5/289 (2%) | PEN: n/a;  TET: 0.25-8;  CFM: 0.015-0.032;  CRO: n/a;  CPD: n/a |
| ........**RD** NVN....--- -G.F..NV.E ..G..Q.... | **III.28** | 1/289 (0.3%) | PEN: n/a;  TET: 2;  CFM: 0.03;  CRO: n/a;  CPD: n/a |
| ........G**D** N.N....--- -G.F..NV.E ..G..K.... | **III.29** | 1/289 (0.3%) | PEN: n/a;  TET: 2;  CFM: 1;  CEF: n/a;  CPD: n/a |
| ........G**S** K.N....--- -G.F..SV.K ..G..E.... | **III.30** | 1/289 (0.3%) | PEN: n/a;  TET: 0.125;  CFM: 0.016;  CRO: n/a;  CPD: n/a |
| ........**ND** N.N....--- -G.F..YV.E ..G..K.... | **III.31** | 1/289 (0.3%) | PEN: n/a;  TET: 2;  CFM: 0.5;  CRO: n/a;  CPD: n/a |

References

1. **Hamilton HL, Dominguez NM, Schwartz KJ, Hackett KT, Dillard JP.** 2005. *Neisseria gonorrhoeae* secretes chromosomal DNA via a novel type IV secretion system. Molecular Microbiology **55:**1704-1721.

2. **Kohler PL, Chan YA, Hackett KT, Turner N, Hamilton HL, Cloud-Hansen KA, Dillard JP.** 2013. Mating pair formation homologue TraG is a variable membrane protein essential for contact-independent type IV secretion of chromosomal DNA by *Neisseria gonorrhoeae*. J Bacteriol **195:**1666-1679.

3. **Ramsey ME, Woodhams KL, Dillard JP.** 2011. The Gonococcal Genetic Island and Type IV Secretion in the Pathogenic *Neisseria*. Frontiers in Microbiology **2:**61.

4. **Rutherford K, Parkhill J, Crook J, Horsnell T, Rice P, Rajandream MA, Barrell B.** 2000. Artemis: sequence visualization and annotation. Bioinformatics **16:**944-945.

5. **Lee EH, Shafer WM.** 1999. The *farAB*-encoded efflux pump mediates resistance of gonococci to long-chained antibacterial fatty acids. Molecular Microbiology **33:**839-845.

6. **Rouquette-Loughlin CE, Balthazar JT, Shafer WM.** 2005. Characterization of the MacA-MacB efflux system in *Neisseria gonorrhoeae*. Journal of Antimicrobial Chemotherapy **56:**856-860.

7. **Lee EH, Rouquette-Loughlin C, Folster JP, Shafer WM.** 2003. FarR regulates the farAB-encoded efflux pump of *Neisseria gonorrhoeae* via an MtrR regulatory mechanism. J Bacteriol **185:**7145-7152.

8. **Rouquette-Loughlin C, Dunham SA, Kuhn M, Balthazar JT, Shafer WM.** 2003. The NorM efflux pump of *Neisseria gonorrhoeae* and *Neisseria meningitidis* recognizes antimicrobial cationic compounds. J Bacteriol **185:**1101-1106.
